# Amber ff24EXP-GA, Based on Empirical Ramachandran Distributions of Glycine and Alanine Residues in Water

**DOI:** 10.1021/acs.jctc.4c01450

**Published:** 2025-02-20

**Authors:** Athul Suresh, Reinhard Schweitzer-Stenner, Brigita Urbanc

**Affiliations:** † Department of Physics, Drexel University, Philadelphia, Pennsylvania 19104, United States; ‡ Department of Chemistry, Drexel University, Philadelphia, Pennsylvania 19104, United States

## Abstract

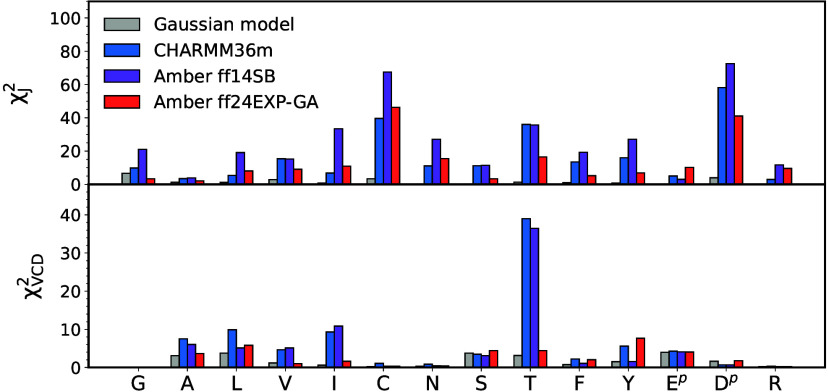

Molecular dynamics (MD) offers important insights into intrinsically disordered peptides and proteins (IDPs) at a level of detail that often surpasses that available through experiments. Recent studies indicate that MD force fields do not reproduce intrinsic conformational ensembles of amino acid residues in water well, which limits their applicability to IDPs. We report a new MD force field, Amber ff24EXP-GA, derived from Amber ff14SB by optimizing the backbone dihedral potentials for guest glycine and alanine residues in cationic GGG and GAG peptides, respectively, to best match the guest residue-specific spectroscopic data. Amber ff24EXP-GA outperforms Amber ff14SB with respect to conformational ensembles of all 14 guest residues x (G, A, L, V, I, F, Y, D^p^, E^p^, R, C, N, S, T) in GxG peptides in water, for which complete sets of spectroscopic data are available. Amber ff24EXP-GA captures the spectroscopic data for at least 7 guest residues (G, A, V, F, C, T, E^p^) better than CHARMM36m and exhibits more amino acid specificity than both the parent Amber ff14SB and CHARMM36m. Amber ff24EXP-GA reproduces the experimental data on three folded proteins and three longer IDPs well, while outperforming Amber ff14SB on short unfolded peptides.

## Introduction

Molecular dynamics (MD) is a robust tool for unraveling the structure and dynamics of proteins, which is often crucial to understanding their biological functions. The reliability of MD predictions, however, depends on the accuracy of the underlying MD force field, which is determined by a complex set of interactions within and among proteins, within and among solvent molecules, and between solvent and protein molecules. Whereas globular proteins with unique folds have been successfully captured by most modern MD force fields, such as Amber ff14SB,^1^ OPLS-AA/M,^2^ and CHARMM36m,^3^ challenges remain when examining structural ensembles of intrinsically disordered peptides and proteins (IDPs).

IDPs make up over 30% of the eukaryotic proteome and play key roles in various biological processes, including human diseases.^4−6^ Their structural plasticity, which allows them to bind multiple partners and perform a variety of biological functions,^7^ makes them difficult to study both experimentally and computationally. Consequently, there is a lack of experimental data on structural constraints on IDPs due to the absence of unique folds and presence of a multitude of conformations that each IDP may adopt upon binding to different partners. Consequently, adequate sampling of the conformational space of an IDP is difficult to achieve in MD simulations. In addition, most IDPs are exposed to the solvent to a much greater extent than globular proteins and need a delicate balance between protein–protein and protein–solvent interactions to be accurately implemented in the MD force field.

MD force fields are known to be highly sensitive to relatively small changes in the backbone dihedral potentials, which directly affect the secondary structure propensities of amino acid residues. This notion led to notable developments in additive MD force fields, culminating in CHARMM36,^8^ OPLS-AA/M,^2^ Amber ff14SB,^1^ and Amber ff19SB,^9^ among many others. These force fields, in which torsional potential parameters were optimized based on quantum chemical calculations on dipeptides or short alanine-based peptides in the gas phase or in implicit solvent, resulted in notable improvements of structure prediction for globular proteins with well-defined folds but predicted overly collapsed structures of IDPs. To address the issue of overly collapsed unfolded or disordered states, Best et al. reported a new force field, Amber ff03ws, based on a modest strengthening of nonspecific water–protein interactions without affecting water–water or protein–protein interactions and showed that this modification leads to improved scaling of unfolded proteins or IDPs with respect to published data obtained by small-angle X-ray scattering and Förster resonance energy transfer.^10^ These and other developments resulted in modifications of CHARMM36 that produced CHARMM36m,^3^ which features reduced sampling of helical conformations, thus avoiding the helical bias of earlier force fields,^11^ and captures experimental data for IDPs better than its predecessor.

Aiming for a force field that would be equally well suited for globular proteins and IDPs, Robustelli et al. assessed a number of MD force fields on a large set of benchmark data and proposed a new force field, a99SB-Disp, derived from Amber ff99SB-ILDN^12^ combined with TIP4P-D water model,^13^ in which torsional potentials and protein–water interactions were optimized based mostly on the available NMR data, *J*-coupling constants, and chemical shifts.^14^ A different strategy to improve the applicability of MD to IDPs was used by Chen and collaborators, who developed a series of new MD force fields based on target Ramachandran distributions derived from protein coil libraries. The resulting force fields include Amber ff99IDPs,^15,16^ Amber ff14IDPs,^17^ CHARMM36IDPSFF,^18^ and Amber ff03CMAP.^19^ A similar coil library-based approach was used by Wu and collaborators, who developed two residue-specific MD force fields, OPLS-based RSFF1^20^ and Amber-based RSFF2.^21^ RSFF1 was among the force fields evaluated by Andrews and collaborators, who showed that although RSFF1 produces amino acid-specific Ramachandran distributions, it does not capture the intrinsic conformational dynamics of amino acid residues in water better than the other force fields, such as OPLS-AA/M and CHARMM36m.^22^ This is most likely because the coil library-derived Ramachandran distribution of a residue of interest can be viewed as a mean-field-like distribution, considering that it is derived as an average over distributions corresponding to various neighboring residues distinctly affecting the conformational ensemble of the residue of interest even in the absence of the secondary structure.^23,24^

We here posit that an MD force field that aims to accurately capture the structure and dynamics of IDPs should be able to reproduce available spectroscopic data that constrain intrinsic conformational ensembles of amino acid residues in water, such as dipeptides or tripeptides. The conformational ensembles of residues in such short peptides are pertinent for IDPs because short peptides are unfolded and exposed to solvent, similar to disordered peptide regions within IDPs. Over the past two decades, Schweitzer-Stenner and collaborators have studied conformational manifolds of guest residues x in cationic GxG peptides in water through the application of solution NMR, vibrational circular dichroism (VCD), Raman, and infrared (IR) spectroscopy, resulting in spectroscopic data comprising five *J*-coupling constants and VCD amide I′ profiles for each of 14 guest residues x (G, A, L, V, I, F, Y, D^p^, E^p^, R, C, N, S, T) in cationic GxG peptides.^25−29^ Each residue-specific set of spectroscopic data can be used as an input to the Gaussian superposition model to obtain the respective Gaussian Ramachandran distribution that best reproduces the experimental constraints.^30^ The resulting residue-specific Gaussian Ramachandran distributions have been used as benchmarks in recent assessment studies of additive MD force fields with respect to their ability to reproduce the above spectroscopic data.^22,31−34^ These studies overall highlighted an important role of water in the stabilization of the polyproline II (pPII) state, which alongside the β-strand-like state dominates the intrinsic conformational ensembles of most guest amino acid residues in water.^31,33,34^ The importance of the water model in MD simulations was further elucidated by demonstrating that replacing TIP3P^35^ by TIP4P-2005^36^ increased the solubility of villin headpiece protein by an order of magnitude.^37^ The more complete assessment of Amber ff14SB, Amber ff19SB,^9^ OPLS-AA/M, and CHARMM36m with respect to capturing experimentally constrained intrinsic conformational ensembles of 14 guest residues x in GxG peptides revealed a severe lack of residue specificity of Ramachandran distributions (with the exception of Amber ff19SB), including a poor reproduction of the residue-to-residue variability of the pPII population and overall large deviations from spectroscopic data for polar and ionizable guest residues.^22^ The benchmark Gaussian Ramachandran distributions outperformed the MD force fields, even CHARMM36m, which reproduced experimental data in many aspects better than the other MD force fields, by at least an order of magnitude.^22^ Additive MD force fields assessed in the above studies are based on classical additive potentials that do not include polarizability effects. One would expect that polarizable MD force fields, such as CHARMM Drude-2019^38,39^ with its intrinsic SWM4-NDP water model^40,41^ and AMOEBA^42^ with its intrinsic AMOEBA 2018 water model^43^ would be more successful in capturing the intrinsic conformational ensembles of amino acid residues in water. Surprisingly, Andrews and collaborators recently demonstrated that the additive MD force field CHARMM36m captures the spectroscopic data for guest glycine and alanine residue in GGG and GAG, respectively, better than either of these two polarizable force fields.^44^

We here propose a minimalistic approach to improve Amber ff14SB by focusing exclusively on optimization of the backbone dihedral potentials for dihedral angles ϕ and ψ, which directly affect the intrinsic conformational ensembles, i.e., the Ramachandran distributions, of amino acid residues in water. For this purpose, we combine Amber ff14SB with the TIP4P-2005 water model. We selected Amber ff14SB because Zhang and collaborators reported that this force field captures the conformational dynamics of the central alanine in cationic GAG and AAA peptides better than CHARMM36m and OPLS-AA force fields.^32^ TIP4P-2005 was chosen based on the results of the assessment study of Amber ff14SB with respect to 14 guest residues x in GxG peptides in water, which showed an overall improvement when TIP3P was replaced by TIP4P-2005.^22^ The overarching aim of this work is to reparametrize the backbone dihedral potentials of the Amber ff14SB force field combined with the TIP4P-2005 water model for only glycine and alanine residues in GGG and GAG, respectively, and demonstrate that the newly optimized MD force field, hereafter referred to as Amber ff24EXP-GA, captures spectroscopic data for all 14 guest residues x in GxG peptides better than the parent Amber ff14SB and offers a moderate improvement over CHARMM36m.

## Methods

### Molecular Dynamics Simulations

Molecular dynamics simulations were performed using GROMACS 5.1.2.^45−51^ The structures of all GxG peptides were prepared using the Visual Molecular Dynamics (VMD) software package.^52^ To match the experimental conditions, the N-terminus was protonated (−NH_3_^+^) and the C-terminus was kept neutral (using C-terminal cappings −COOH in CHARMM36m and −CO–NH_2_ in Amber). The peptides were solvated in a cubic box with an edge length of 5 nm using the intrinsic TIP3P water model for CHARMM36m simulations and the TIP4P-2005 water model for Amber simulations. One or two (for GRG) chloride ions were added to neutralize the system. The solvated system was energy minimized using the steepest descent algorithm until the maximum force was lower than the value of 1000.0 kJ mol^–1^ nm^–1^ with a step size of 0.01 nm and a cutoff distance of 1.0 nm for both van der Waals and electrostatic interactions, as recommended for Amber ff14SB by Maier and collaborators.^1^ For simulations with the CHARMM36m force field, the cutoff distance was 1.2 nm instead, as recommended for this force field in GROMACS documentation. The integration time step of 2 fs was used in all simulations. Long range electrostatic interactions were computed using the Particle Mesh Ewald (PME)^53^ method with an order of 4 and grid spacing equal to 0.16 nm. Following energy minimization, the system was equilibrated at 300 K with a 1 ns long NVT equilibration using the stochastic V-rescale thermostat,^54^ followed by a 1 ns long NPT equilibration using the Parrinello–Rahman barostat^55^ with time constants τ_*T*_ and τ_*P*_ equal to 1.0 and 5.0 ps, respectively, and an isothermal compressibility of 4.5 × 10^–5^ bar^–1^. During the equilibration step, the peptide coordinates were restrained using a harmonic force with force constant of 1000.0 kJ mol^–1^ nm^–1^, and the bonds involving hydrogen atoms were constrained using the LINCS algorithm.^48^ Production runs, each 500 ns long, were performed using the same thermostat and barostat used during the equilibration. The coordinates were saved every 10 ps. Simulations of cationic GGG peptides in Amber ff0ws with TIP4P-2005 water and Amber 99SB-Disp with TIP4P-D water were set up in the same way as the Amber ff14SB simulations described above, with the nonbonded cutoff distances set to 1.2 and 1.4 nm, respectively, as recommended by the developers of these two force fields.^10,14^ CHARMMIDPSff simulations with TIP3P water followed the same protocol as the CHARMM36m simulations described above.

We also examined six longer peptides and proteins: (i) chingnolin (CLN025) with amino acid sequence YYDPETGTWY and experimentally resolved structure (PDB-ID: 2RDV),^56^ (ii) villin headpiece (HP36) with amino acid sequence MLSDEDFKAVFGMTRSAFANLPLWKQQNLKKEKGLF and experimentally resolved structure (PDB-ID: 1VII),^57^ (iii) third immunoglobin binding domain of protein G (GB3) with amino acid sequence MQYKLVINGKTLKGETTTKAVDAETAEKAFKQYANDNGVDGVWTYDDATKTFTVTE and experimentally resolved structure (PDB-ID: 1P7E),^58^ (iv) pentaalanine (Ala_5_) with multiple reported experimental *J*-coupling constants,^59^ (v) histatin 5 with amino acid sequence DSHAKRHHGYKRKFHEKHHSHRGY and previously reported experimental per-residue *J*-coupling constant values,^60^ and (vi) Aβ40 with amino acid sequence DAEFRHDSGYEVHHQKLVFFAEDVGSNKGAIIGLMVGGVV with previously reported experimental per-residue *J*-coupling constant values.^61^ To this end, we acquired three 1 μs long MD trajectories for each system and each of the three MD force fields, CHARMM36m, Amber ff14SB, and Amber ff24EXP-GA, amounting to 6 × 3 × 3 × 1 μs = 54 μs of simulations. To match the experimental conditions, aspartic acid, glutamic acid, and histidine residues as well as ionizable −COOH groups were protonated. For the three proteins with reported folded structure (i–iii), RMSD analysis was performed using all time frames, separated by 10 ps, of the three replica trajectories per system. The average coil, strand, turn, and helical content for systems i–iii, derived from the secondary structure assignments using the STRIDE algorithm^62^ in *Visual Molecular Dynamics*,^52^ were calculated as averages over the time interval between 50 and 1000 ns, separated by 1 ns, and over three replica MD trajectories per system. For the three IDPs (iv–vi), the *J*-coupling constants were calculated as averages over time frames, separated by 10 ps, along the entire 1 μs long trajectory and over the three replica trajectories per system.

### Iterative Boltzmann Inversion

Our strategy to improve the backbone dihedral potentials of Amber ff14SB combined with the TIP4P-2005 water model consists of the iterative Boltzmann inversion (IBI) method, followed by an *ad hoc* rescaling of the potentials to obtain minimal magnitudes of the dihedral potentials with desired outcomes. The IBI method is a statistical mechanics-based algorithm for calculating the potential energy of the system from known probability distributions and involves self-consistently adjusting a given potential to achieve convergence with the target probability distribution.^63,64^ In our work, the potential term of interest is the Amber backbone dihedral potential *V*(ϕ, ψ), and the target probability distribution is the residue-specific Gaussian model-based Ramachandran distribution for guest residue x in the GxG peptide, which is constructed to best fit the spectroscopic data. In this work, we focus specifically on the optimization of backbone dihedral potentials for guest glycine and alanine residues in GGG and GAG peptides, respectively.

In Amber force fields, the backbone dihedral potentials in ϕ and ψ are approximated by a sum of two Fourier series for each of the dihedral angles:
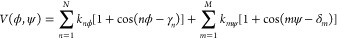
1where *N* and *M* correspond to the highest-order nonzero terms in Fourier expansions. In Amber ff14SB, *N* = 3 and *M* = 3. In our IBI optimization of the dihedral potential *V*(ϕ, ψ), the number of terms is increased to *N* = *M* = 5 to better fit the target Gaussian Ramachandran distribution.

The IBI method consists of an iterative procedure, whereby at each step *i*, we derive the potential of the mean force (free energy) difference between MD-derived and Gaussian Ramachandran distributions:
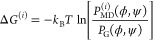
2where *P*_G_(ϕ, ψ) is the target Gaussian Ramachandran distribution, *P*_MD_^(*i*)^(ϕ, ψ) is the distribution derived from MD at iteration step *i* using the potential *V*^(*i*)^(ϕ, ψ).

The initial parameters, {*k*_*n*ϕ_^(0)^} and {*k*_*m*ψ_^(0)^}, which define the initial dihedral potential *V*_MD_^(0)^(ϕ, ψ) = 0, are set to zero. At each iteration step (*i* + 1), we obtain the new potential *V*_MD_^(*i*+1)^(ϕ, ψ) by performing MD simulations of the respective GxG peptide in water using dihedral potential *V*^(*i*)^(ϕ, ψ) and then extracting the Ramachandran distribution (using the gmx rama module of GROMACS) for guest residue x, *P*_MD_^(*i*)^(ϕ, ψ), resulting in
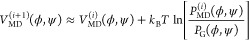
3The new set of parameters ({*k*_*n*ϕ_^(*i*+1)^}, {*k*_*m*ψ_^(*i*+1)^}) is obtained by fitting the potential form in eq 1 to the term Δ*V*_MD_^(*i*+1)^ = *V*_MD_^(*i*+1)^ – *V*_MD_^(*i*)^, to obtain the change in dihedral parameters Δ*k*_*n*ϕ_^(*i*)^ and Δ*k*_*m*ψ_^(*i*)^.

The dihedral parameters changes, Δ*k*_*n*ϕ_^(*i*)^ and Δ*k*_*m*ψ_^(*i*)^, were obtained by minimizing the loss function
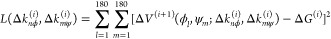
4where dihedral angle space was discretized as ϕ_*l*_ = −179°, −177°, ..., 177°, 179° and ψ_*m*_ = −179°, −177°, ..., 177°, 179°. The minimization was performed using the conjugate gradient descent method,^65^ using the pytorch python library.

**Figure 1:**
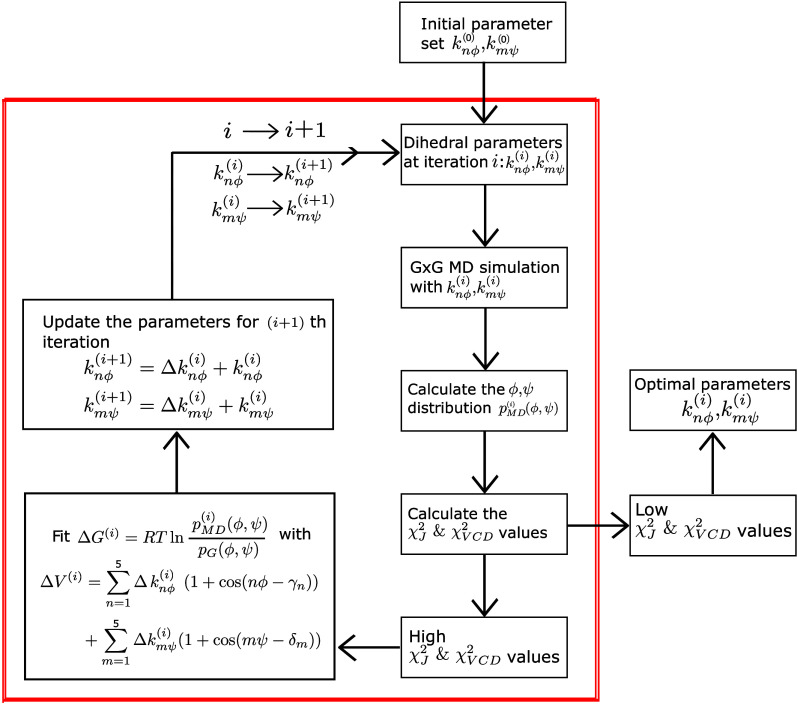
Parametrization procedure using the Iterative Boltzmann Inversion (IBI) method.

The parameters for the next iteration are obtained as
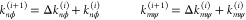
5The iterative process is schematically depicted in Figure 1.

### Comparison to Spectroscopic Data

Conformational ensembles of short unfolded peptides can be determined using various spectroscopic methods, which are sensitive to the backbone dihedral angles ϕ and ψ. In this work, we use five *J*-coupling constants, ^3^*J*(H^N^, H^C_α_^), ^3^*J*(H^N^, C′), ^3^*J*(H^C_α_^, C′), ^3^*J*(H^N^, C_β_) (for all guest residues other than glycine) or ^3^*J*(C, C′) (for guest glycine residue in GGG), and ^1^*J*(N, C_α_), and amide I′ profiles corresponding to IR, VCD, and Raman spectra of the guest residue x in GxG peptides. A direct comparison with experimental data can be made by calculating the experimental observable
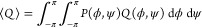
6where *Q* is either a *J*-coupling constant or amide I′ intensity (the prime sign in I′ indicates that the measurements were performed in D_2_O), both of which are sensitive to the backbone dihedral angles.

An extensive set of experimental *J*-coupling constants for the guest residue x in cationic GxG peptides, namely, ^3^*J*(H^N^, H^C_α_^), ^3^*J*(H^N^, C′), ^3^*J*(H^N^, C_β_) (for all guest residues other than glycine) or ^3^*J*(C, C′) (for guest glycine residue in GGG), ^3^*J*(H^N^, C_α_), and ^1^*J*(N, C_α_), were reported in prior works.^25−28,66^ These *J*-coupling constants are sensitive to backbone dihedral angles ϕ and ψ (only ^1^*J*(N, C_α_)) and can be computed using eq 6, where the *J*-coupling constant is the observable *Q* and depends on the dihedral angle through the Karplus equation^67^
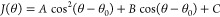
7

The constants *A*, *B* and *C* are Karplus parameters which are obtained mostly from plots of experimental *J*-coupling constants of residues in folded proteins, for which X-ray crystallographic and/or NMR-based structures are available. For the alanine residue, Karplus constants obtained from density functional theory calculations are also available.^68^ In line with earlier works,^25,26,59^ we utilized the Karplus parameters as well as the phases θ_0_ from the paper of Hu and Bax^69^ for all ϕ-dependent ^3^*J*-coupling constants. For ^1^*J*(N, C_α_), we used the Karplus parameters reported by Wirmer and Schwalbe.^70^

### The Gaussian Model

The Gaussian superposition model uses 2D Gaussian subdistributions to describe residue-specific conformational ensembles associated with several typical mesostates, such as pPII, parallel and antiparallel β-strand, transitional β, helical, and other turn-like states in the Ramachandran space.^30^ The weighted sum of these Gaussian subdistributions gives rise to the Gaussian Ramachandran distribution, i.e., the probability distribution in the phase space of the two backbone dihedral angles, *P*(ϕ, ψ):
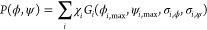
8where χ_*i*_ is the weight (mole fraction) of the *i* mesostate, modeled by a two-dimensional Gaussian distribution *G*_*i*_ located at ϕ_*i*, max_ and ψ_*i*, max_ with half-widths σ_*i*,ϕ_ and σ_*i*,ψ_. For any given dihedral angle distribution, *P*(ϕ, ψ), the *J*-coupling constants are calculated using Karplus equations with previously reported Karplus parameters.^59^ The corresponding amide I′ band profiles are derived for ϕ and ψ values and averaged over the ϕ−ψ space. As described in the previous section, the parameters of the Gaussian model (χ_*i*_ values, positions, and widths of the corresponding Gaussian distributions) are then adjusted in such a way that the calculated *J*-coupling constants and amide I′ profiles best match the corresponding experimental data.

### Definition of Mesostates

**Figure 2:**
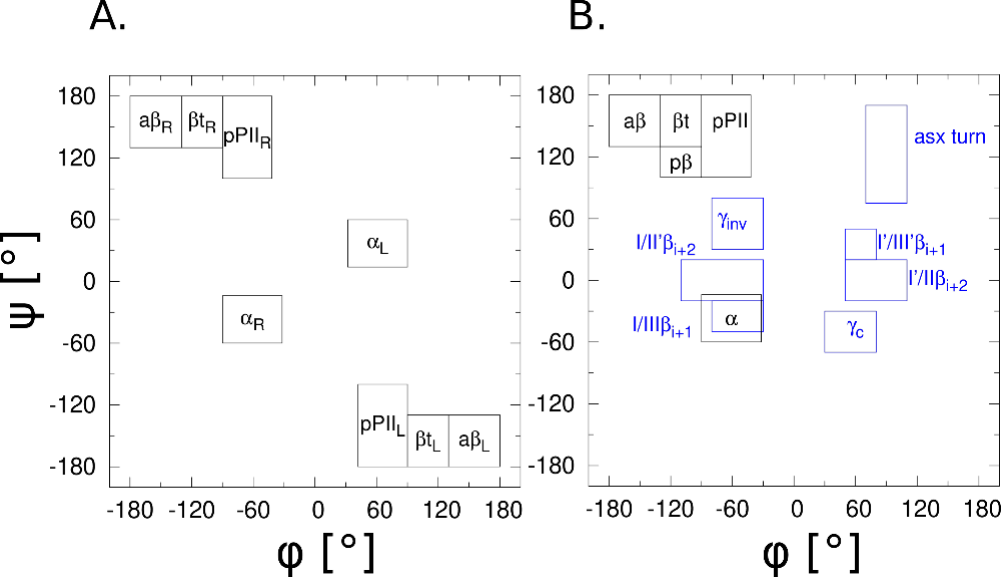
Definitions of the four mesostates in the Ramachandran plot for (A) GGG (achiral) and (B) GxG (chiral) systems. The labels aβ, pβ, βt, pPII, and α correspond to antiparallel β, parallel β, transitional β, polyproline II, and right-handed helix, respectively. The turn-supporting mesostates are marked blue.

Consistent with the recent study,^22^ we use the following mesostate definitions: (a) polyproline II, pPII (−90° < ϕ < −42°, 100° < ψ < 180°), (b) antiparallel β-strand, aβ (−180° < ϕ < −130°, 130° < ψ < 180°), (c) β transition region between aβ and pPII, βt (−130° < ϕ < −90°, 130° < ψ < 180°), (d) right-handed helix, α (−90° < ϕ < −32°, −60° < ψ < −14°). The mesostate definitions for the achiral guest glycine and chiral residues are shown in Figure 2A,B, respectively. The four mesostates that dominate the conformational ensembles of most guest residues x in GxG peptides (pPII, aβ, βt, and right-handed helix) are marked as black rectangles in Figure 2B and are displayed on all Ramachandran distributions in this work as guides to the eye. Conformations that support turn-like structures are type I/II′ β_*i*+2_ (−110° < ϕ < −30°, −20° < ψ < 20°), type I′/II β_*i*+2_ (50° < ϕ < 110°, −20° < ψ < 20°), and asx (70° < ϕ < 110°, 75° < ψ < 170°), shown as blue rectangular regions in Figure 2B.

### MD-Derived Ramachandran Distributions

Ramachandran distributions are constructed from dihedral angles (ϕ, ψ) within GROMACS 5.1.2 using 25000 time frames of each MD trajectory starting from 50 ns (with time frames 2 ps apart). Normalized 2D distributions are calculated with a bin size of 2° × 2°, resulting in 180 × 180 = 32400 bins in the range −179, −177, ..., 177, 179 along the ϕ and ψ coordinates to facilitate a direct comparison to the Ramachandran distributions predicted by the Gaussian model.

### Hellinger Distance

The Gaussian and MD-derived Ramachandran distributions are also used to calculate the Hellinger distance^71^ between Ramachandran distributions of residues A and B:
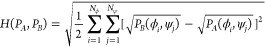
9where *P*_*A*_(ϕ_*i*_, ψ_*j*_) and *P*_*B*_(ϕ_*i*_, ψ_*j*_) are the probabilities corresponding to bin of index *ij* in the discretized ϕ, ψ space, where ϕ is divided into *N*_ϕ_ bins and ψ to *N*_ψ_ bins.

### Calculation of *J*-Coupling Constants and Amide I′ Profiles from MD Data

We use MD-derived Ramachandran distributions to calculate the *J*-coupling constants and amide I′ profiles for guest residue x in cationic GxG peptides. With regard to *J*-coupling constants, we characterize the overall difference between the calculated and experimental values by the absolute value of their differences as well as by the χ_*J*_^2^ function:
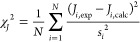
10where *J*_*i*,exp_ and *J*_*i*,calc_ denote experimental and calculated *J*-coupling constant values (obtained by using eqs 6 and 7), respectively, *N* is the number of *J*-coupling constants, and *s*_*i*_ are statistical uncertainties, i.e., combinations of experimental errors^25^ and errors due to the reported errors in the estimation of the Karplus parameters.^69^ Statistical uncertainties *s*_*i*_ are calculated as ensemble averages of the following function *s̅*_*i*_(θ):
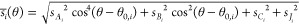
11where *s*_*A*_*i*__, *s*_*B*_*i*__, *s*_*C*_*i*__ are the statistical uncertainties associated with the three Karplus parameters, θ and θ_0,*i*_ are the relevant dihedral angle and phase associated with the *J*-coupling constant *J*_*i*_, respectively, and *s*_*J*_*i*__ is the corresponding experimental uncertainty. The statistical uncertainties of the three Karplus parameters are available for all *J*-coupling constants of guest residues x in GxG peptides used in this work except for ^1^*J*(N, C_α_).

The accuracy with respect to the reproduction of the VCD band profile was compared using
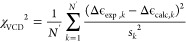
12where Δϵ represents the molar dichroism and *k* labels the wavenumber index. *N*′ is the number of data points considered, and the standard error *s*_*k*_ is derived from the analysis of a spectral region dominated by noise.^30^ Experimental molar dichroism values, Δϵ_exp,*k*_, were calculated by employing the Beer–Lambert law as reported previously.^25−27,72^ The corresponding VCD profiles were calculated by employing the coupled oscillator approach described in earlier work.^30,73^

## Results and Discussion

The overarching aim of this work is to develop a new Amber ff14SB-based force field with improved intrinsic conformational dynamics for 14 amino acid residues x in GxG peptides in water as assessed by five experimentally derived *J*-coupling constants (see Methods) and VCD amide I′ profiles per residue. In our force field optimization strategy outlined below, we use the TIP4P-2005 water model with the original Amber force field ff14SB as well as with the newly optimized Amber ff24EXP-GA.^36,74,75^ This water model when used in Amber ff14SB showed a small overall improvement in the Ramachandran distributions of several guest residues x in GxG peptide over TIP3P^22^ and was reported to increase the solubility of villin headpiece by 1 order of magnitude, in line with experimental data.^37^ Andrews and collaborators also reported on insufficient specificity of guest residue Ramachandran distributions shared by Amber ff14SB, CHARMM36m, and OPLS-MM.^22^ Replacing TIP3P by TIP4P-2005 alone did not increase the specificity of guest residue Ramachandran distributions in Amber ff14SB.^22^

The optimization strategy uses the experimentally constrained guest residue-specific Ramachandran distributions that have been modeled as a set of normal subdistributions for each of the typically populated basins in the Ramachandran space: pPII, antiparallel β-strand (aβ), transitional β (βt), and helical (α) (Figure 2), as well as various turns, such as asx, γ, and inverse γ. The parameters of these normal distributions have then been optimized to best fit the spectroscopic data for each guest residue x in GxG.^30^ In the following, we refer to these Ramachandran distributions as Gaussian distributions and use them both in the force field optimization and as a benchmark for comparison of MD-derived spectroscopic data with experimental data.

Amber ff24EXP-GA is based on the optimization of backbone dihedral potentials of Amber ff14SB, i.e., dihedral potential *V*(ϕ, ψ), which is applied to all amino acid residues, and *V*(ϕ′, ψ′), which is applied to all non-glycine residues. In Amber ff24EXP-GA, the latter potential, *V*(ϕ′, ψ′), is set to zero except for proline, for which the original Amber ff14SB parameters are applied. This choice is motivated by the empirical observation that setting *V*(ϕ′, ψ′) to zero increases the guest residue specificity of the resulting Ramachandran distributions. It is unclear why this is so, but it appears that a nonzero *V*(ϕ′, ψ′) introduces conformational restrictions that limit the flexibility of the side chain relative to the backbone. In Amber ff14SB, *V*(ϕ, ψ) = *V*_ϕ_(ϕ) + *V*_ψ_(ψ), each dihedral potential is a series of cosine functions, and only three terms are used in each series. We explored the expansion of the cosine series first into four and then into five terms, for both guest glycine and alanine in GxG peptides, to determine how many terms are needed to capture the experimental data as well as the Gaussian model. The results of the application of the IBI method to four and five terms are displayed in Figure S3. Close examination of *J*-coupling constants for guest glycine in GGG shows that both four- and five-term expansions produce similar results with respect to producing χ_*J*_^2^ that is comparable to the benchmark Gaussian model. On the other hand, the five-term expansion produced lower χ_*J*_^2^ and χ_VCD_^2^ values for guest alanine in GAG than the four-term expansion. We thus expanded these potentials to include five terms in each series. Our optimization strategy outlined below consists of simultaneous optimization of *V*_G_(ϕ, ψ) and *V*_A_(ϕ, ψ) for the guest glycine residue in GGG and guest alanine residue in GAG, respectively. The convergence of the potential parameters for both guest glycine and alanine in GGG and GAG, respectively, is shown in Figure S3.

### Optimization of the Backbone Dihedral Parameters for the Guest Glycine Residue in Cationic GGG

**Figure 3:**
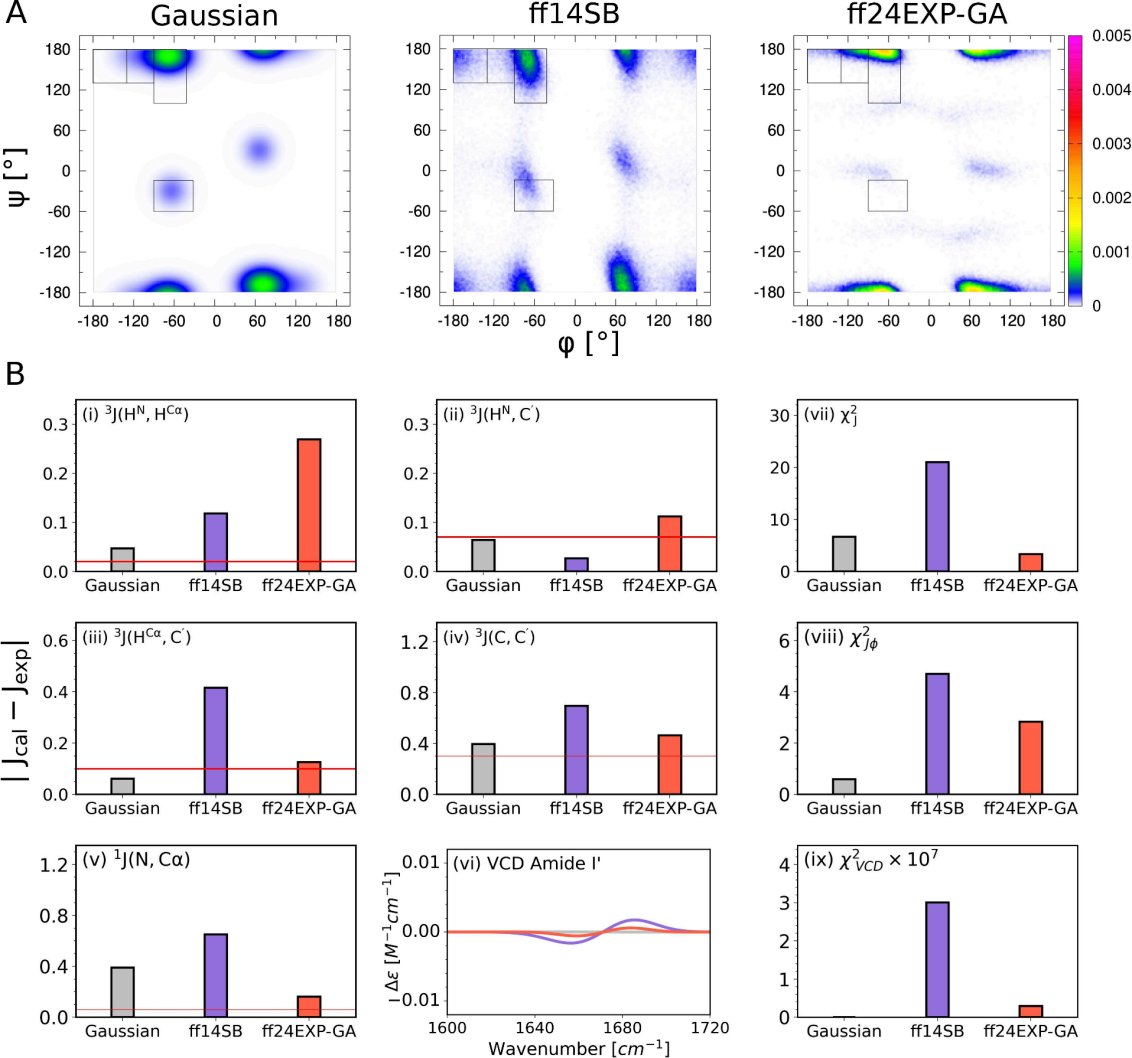
(A) Ramachandran distributions of guest glycine in cationic GGG for the experiment-based Gaussian model (benchmark data), the parent Amber ff14SB, and the newly modified Amber ff24EXP-GA. (B) Comparison between experimental and computed *J*-coupling constants and amide I′ profiles of guest glycine in GGG for the Gaussian model and Amber ff14SB and Amber ff24EXP-GA. (i–v) Absolute differences between calculated and experimental values of the five *J*-coupling constants for the Gaussian model and the two MD force fields. Red lines correspond to experimental uncertainties. (vi) VCD amide I′ profiles computed using the Gaussian model and the two MD force fields in comparison to experimental data. (vii–ix) Reduced χ_*J*_^2^, χ_*J*ϕ_^2^ and χ_VCD_^2^ values for the Gaussian model and the two MD force fields. χ_VCD_^2^ values are multiplied by 10^7^.

We applied the iterative Boltzmann inversion (IBI) method (see Methods) to modify the backbone dihedral potentials for the guest glycine residue in cationic GGG, where the initial Ramachandran distribution was obtained from Amber ff14SB with *V*(ϕ, ψ) = 0 and the target Ramachandran distribution was the Gaussian distribution (Figure 3A, left panel). We used a combination of the IBI and manual rescaling to arrive at the optimal parameter set for glycine as graphically shown in Figure S4. The corresponding optimized potentials *V*_ϕ_ and *V*_ψ_ are displayed in Figure S3, which also shows the corresponding original Amber ff14SB potentials for comparison. Figure 3A shows the Ramachandran distributions for the guest glycine of the Gaussian model (left plot), Amber ff14SB (middle plot), and the Amber force field with the newly optimized backbone dihedral parameters, which we will refer to hereafter as the Amber ff24EXP-GA force field. Figure 3B compares the spectroscopic data for the guest glycine in GGG to the respective calculated *J*-coupling constants and VCD amide I′ profiles derived from the Ramachandran distributions of the Gaussian model, Amber ff14SB, and Amber ff24EXP-GA. The absolute differences between the five calculated and experimental *J*-coupling constants, shown in Figure 3B(i–v), demonstrate that Amber ff24EXP-GA results in an improvement for 3 out of 5 *J*-coupling constants considered here. The better accuracy in capturing ^1^*J*(N, C_α_) is noteworthy because this *J*-coupling constant is the only one of the five that depends on ψ and is thus sensitive to the location of the mesostates in the Ramachandran distribution along the ψ coordinate. The VCD amide I′ profile (Figure 3B(vi)) should be identically equal to zero due to the achiral nature of GGG. Amber ff14SB and Amber ff24EXP-GA result in nonzero VCD peaks; however, their magnitudes are below the experimental detection level. The overall improved accuracy in capturing the *J*-coupling constants is reflected in χ_*J*_^2^ and χ_*J*ϕ_^2^ (see Methods) for all five *J*-coupling constants and the four ϕ-dependent *J*-coupling constants, respectively, which are significantly lower for Amber ff24EXP-GA than for Amber ff14SB (Figure 3B(vii,viii)).

### Optimization of the Backbone Dihedral Parameters for the Guest Alanine Residue in Cationic GAG

**Figure 4:**
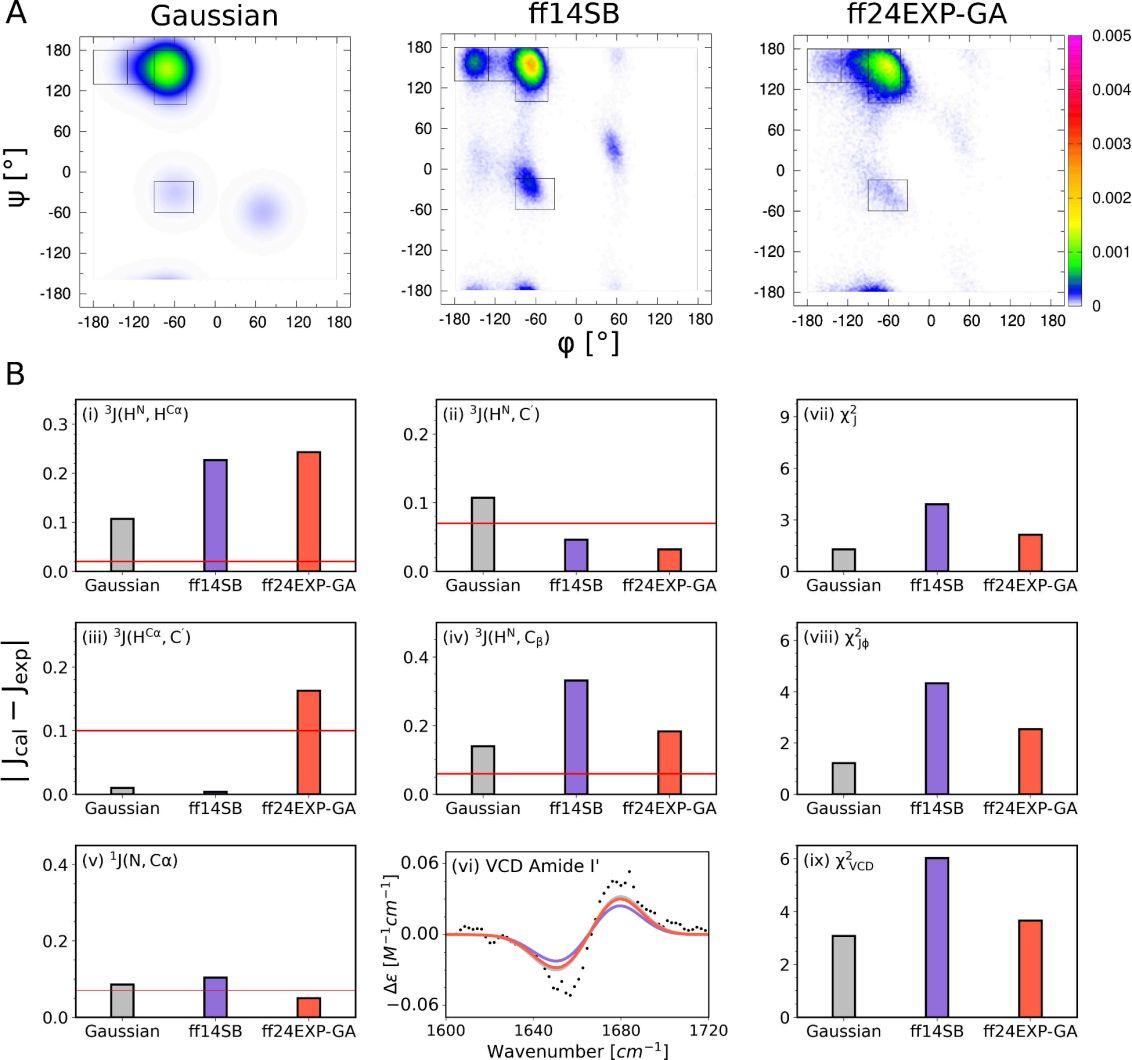
(A) Ramachandran distributions of guest alanine in cationic GAG for experiment-based Gaussian model (left), Amber ff14SB (middle), and Amber ff24EXP-GA (right). (B) Comparison between experimental and computed *J*-coupling constants and amide I′ profiles of guest alanine in GAG for the Gaussian model and Amber ff14SB and Amber ff24EXP-GA. (i–v) Absolute differences between calculated and experimental values of the five *J*-coupling constants for the Gaussian model and the two MD force fields. Red lines correspond to experimental uncertainties. (vi) VCD amide I′ profiles computed using the Gaussian model and the two MD force fields in comparison to experimental data. (vii–ix) Reduced χ_*J*_^2^, χ_*J*ϕ_^2^ and χ_VCD_^2^ values for the Gaussian model and the two MD force fields.

Except for glycine, alanine is the simplest amino acid residue with a methyl group as the side chain. Due to the presence of a C_β_ carbon, there are two additional dihedral potential terms in Amber ff14SB that apply to alanine and other non-glycine residues. In addition to the dihedral angles ϕ (C–N–C_α_–C) and ψ (N–C_α_–C–N), non-glycine residues are described in addition by angles ϕ′ (C–N–C_α_–C_β_) and ψ′ (C_β_–C_α_–C–N), resulting in the additional dihedral potential *V*(ϕ′, ψ′), which is set to zero in Amber ff24EXP-GA. The Gaussian model Ramachandran distribution of the alanine residue in GAG differs significantly from that of the guest glycine residue in GGG, not just due to the absence of inversion symmetry about the origin, ϕ, ψ = (0°, 0°), but also with respect to the width and location of the pPII basin, which is broader and shifted to lower ψ values in comparison to the right-handed pPII basin of glycine residue in GGG (Figure 4A, left panel). The optimal dihedral parameters derived for glycine (in the previous section) do not capture the Ramachandran distribution of the alanine residue accurately enough (Figure S6A). Hence, we sought to optimize the dihedral parameters for the alanine residue in GAG by applying the IBI method. As shown in Figure S6B, the optimal backbone dihedral parameters with the Gaussian model for the alanine residue in GAG as the reference distribution are obtained after a single step of IBI, starting from the Ramachandran distribution derived for the alanine residue in GAG from Amber ff14SB simulations, in which both backbone dihedral potentials were set to zero. The resulting optimized dihedral potentials (Amber ff24EXP-GA) are plotted in Figure S5 for comparison. Figure 4A shows the Ramachandran distributions for the guest alanine of the Gaussian model (left), Amber ff14SB (middle), and the Amber ff24EXP-GA force field (right). Figure 3B compares the spectroscopic data for the guest alanine in GAG to the respective calculated *J*-coupling constants and VCD amide I′ profiles derived from the Ramachandran distributions of the Gaussian model, Amber ff14SB, and Amber ff24EXP-GA. The absolute differences between the five calculated and experimental *J*-coupling constants, shown in Figure 4B(i–v), demonstrate that Amber ff24EXP-GA results in improvement for 3 out of 5 *J*-coupling constants considered here. In the case of GAG in addition to the ^1^*J*(N, C_α_) constant, the VCD amide I′ profile (Figure 4B(vi)) is also dependent on ψ, and both these observables are thus sensitive to the location of the populations mesostates in the Ramachandran distribution along the ψ coordinate. The overall improved accuracy in capturing the *J*-coupling constants is reflected in χ_*J*_^2^ and χ_*J*ϕ_^2^ for all five *J*-coupling constants and the four ϕ-dependent *J*-coupling constants, respectively, which are significantly lower for Amber ff24EXP-GA than for Amber ff14SB (Figure 4B(vii,viii)). The VCD amide I′ profile and the corresponding χ_VCD_^2^ predicted by Amber ff24EXP-GA are also improved over the respective Amber ff14SB values (Figure 4B(vi,ix)). The newly optimized backbone dihedral potentials for alanine residue in Amber ff24EXP-GA are displayed in Figure S5 to allow for a comparison to the respective potentials in Amber ff14SB.

### Setting Up Dihedral Potentials for 20 Amino Acid Residues in Amber ff24EXP-GA

After deriving two different sets of backbone dihedral potentials *V*_G_(ϕ, ψ) and *V*_A_(ϕ, ψ) for glycine and alanine, guided by spectroscopic data for cationic GGG and GAG, respectively, we asked which one of the two potentials would be more accurate in capturing the experimentally obtained conformational propensities of guest residues x in 12 GxG peptides, namely x = L, V, I, F, Y, C, N, S, T, D^p^, E^p^, and R, for which complete sets of spectroscopic data are available. To this end, MD simulations of these 12 GxG peptides were performed using Amber ff14SB with TIP4P-2005 with modified backbone dihedral potentials *V*_G_(ϕ, ψ) and *V*_A_(ϕ, ψ), henceforth termed Amber ff24EXP-G and Amber ff24EXP-A, respectively. The resulting Ramachandran distributions are shown in Figures S7–S10 and the corresponding χ_*J*_^2^, χ_*J*ϕ_^2^, and χ_VCD_^2^ values for Gaussian model, Amber ff14SB, and the two new parametrizations, Amber ff24EXP-G and Amber ff24EXP-A, are compared in Figure S11. Both qualitative and quantitative comparisons of Ramachandran distributions and χ^2^ values demonstrate that the conformational ensembles of the 8 guest residues V, I, F, Y, S, T, D^p^, and E^p^ are better captured by Amber ff24EXP-G than by Amber ff24EXP-A, whereas Amber ff24EXP-A reproduces the remaining guest residues, L, C, N, and R, better than Amber ff24EXP-G. We thus designate the former 8 guest residues as “glycine-like” or G-like and the remaining 4 guest residues as “alanine-like” or A-like (Table S3). The fully optimized force field Amber ff24EXP-GA is then defined as a combination of Amber ff24EXP-G and Amber ff24EXP-A, in which only two types of backbone dihedral parameters are used, *V*_G_(ϕ, ψ) for V, I, F, Y, S, T, D^p^, and E^p^ and *V*_A_(ϕ, ψ) for L, C, N, and R.

The complete sets of spectroscopic data are not available for the following residues: H, W, M, Q, and K. To complete the definition of Amber ff24EXP-GA, i.e., select the backbone dihedral potential parameters for these remaining 5 amino acid residues, we assigned to each of them *V*_G_(ϕ, ψ) or *V*_A_(ϕ, ψ) in an *ad hoc* fashion, based on structural and/or chemical side-chain similarities to the previously assigned G-like or A-like residues. Aromatic H and W with sterically bulkier rings connected to the C_β_ carbon are treated as G-like in analogy to F and Y. Amino acid residues K, M, and Q which are not branched at C_β_ are treated as A-like (see Table S3). All backbone dihedral parameters for P are directly imported from Amber ff14SB. The new force field Amber ff24EXP-GA parameters formatted for GROMACS are provided as a zip folder in the Supporting Information.

### Evaluation of Amber ff24EXP-GA with Respect to Guest Residues x in Cationic GxG Peptides

Andrews and collaborators have shown that one of the limitations of the three contemporary MD force fields, Amber ff14SB, OPLS-AA/M, and CHARMM36m, is their inability to capture the amino acid residue specificity of the intrinsic conformational ensembles as assessed through spectroscopic data on guest residues x in cationic GxG peptides in water.^22^ We here test the new force field, Amberff24EXP-GA, with reparametrized backbone dihedral potentials on the 12 GxG peptides with guest residues x = L, V, I, F, Y, C, N, S, T, D^p^, E^p^, and R. To facilitate the comparison, we categorize the amino acid residues into 4 groups, based on the chemical nature of their side chains, and compare the conformational ensembles of Amber ff24EXP-GA to those derived in Amber ff14SB and CHARMM36m. Both Amber force fields are combined with TIP4P-2005 water model, whereas CHARMM36m uses its own modified TIP3P water model.

#### Aliphatic Amino Acid Residues: L, V, and I

**Figure 5:**
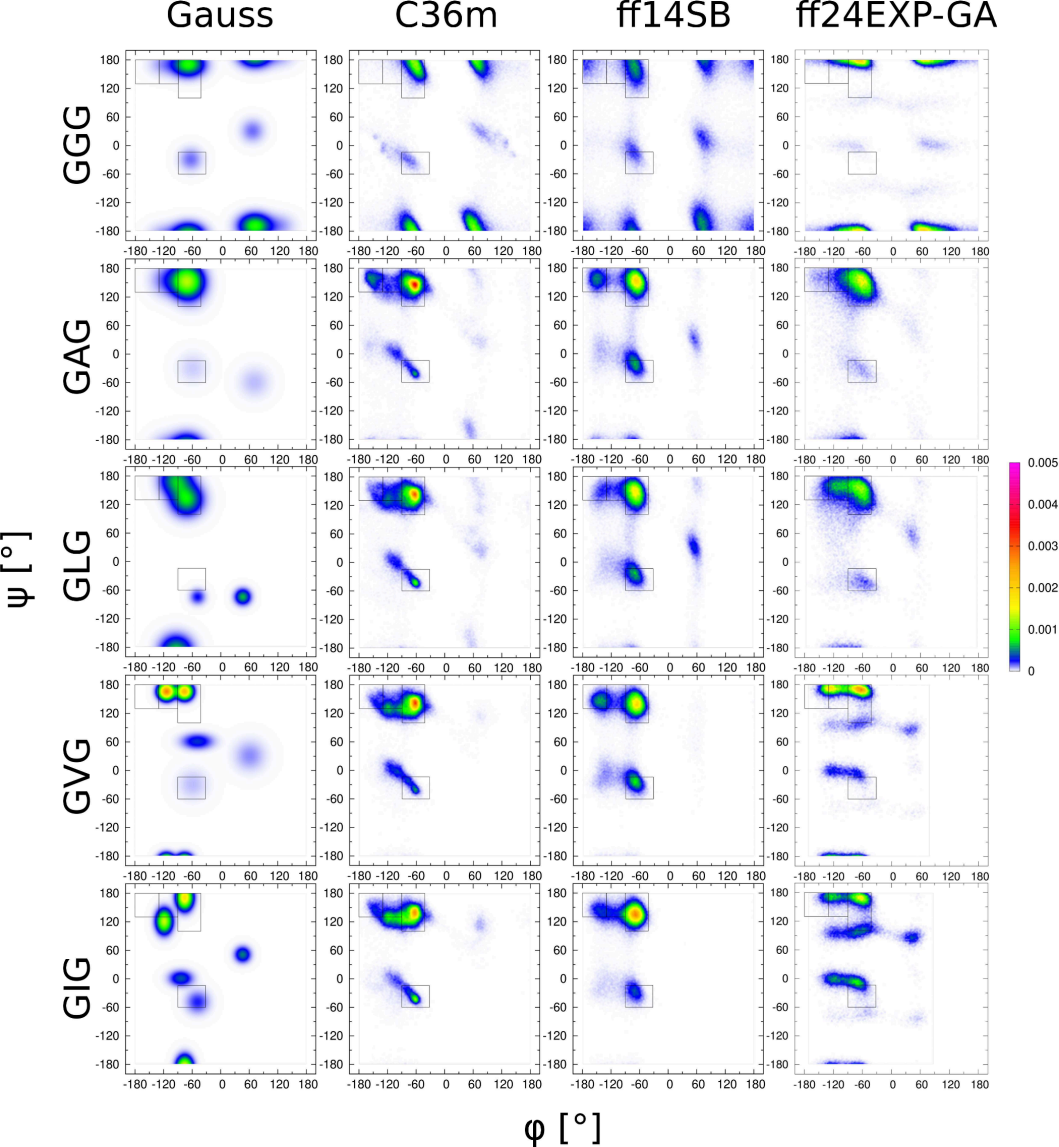
Ramachandran distributions of all aliphatic guest amino acid residues GxG, x = G, A, L, V, I, for different MD force fields assessed and the Gaussian model.

The Ramachandran distributions for G, A, L, V, and I derived from the Gaussian model of the three MD force fields are shown in Figure 5. The Gaussian Ramachandran distributions of these five guest residues vary significantly with respect to each other. In contrast, the Ramachandran distributions for these five guest residues are almost identical in CHARMM36m and Amber ff14SB, although there are significant differences between these two force fields. The new force field Amber ff24EXP-GA exhibits significantly more residue specificity in the Ramachandran distributions than Amber ff14SB and CHARMM36m. Importantly, the accuracy of Amber ff24EXP-GA in capturing the *J*-coupling constants and VCD amide I′ profiles for L, V, and I is improved over Amber ff14SB as well as CHARMM36m as shown in Figures S12–S14.

A comparison to experimental data for leucine in GLG in Figure S12 shows that Amber ff24EXP-GA outperforms Amber ff14SB with respect to *J*-coupling constants, resulting in lower χ_*J*ϕ_^2^ and χ_*J*_^2^ values, but is not improved over CHARMM36m. Amber ff24EXP-GA results in a χ_VCD_^2^ value comparable to that of Amber ff14SB and significantly lower than that of CHARMM36m. The Ramachandran distribution for valine in GVG is significantly different from the one for leucine in GLG, as reflected in the corresponding Gaussian models (Figure 5) and shows a greater propensity for the βt conformation at the expense of pPII. Unlike CHARMM36m and Amber ff14SB, which do not capture these conformational changes,^22^ Amber ff24EXP-GA reproduces this trend. The balance between β and pPII conformations is dictated by dihedral angle ϕ, and on comparing the quantities χ_*J*ϕ_^2^ and χ_VCD_^2^, which depend on ϕ, Amber ff24EXP-GA exhibits the lowest values among the compared force fields as shown in Figure S13 (viii,ix). However, for ^1^*J*(N, C_α_), which depends only on dihedral angle ψ, Amber ff24EXP-GA deviates from the experimental data more than Amber ff14SB and CHARMM36m. Isoleucine is branched at the C_β_ carbon and has conformational propensities similar to that of valine, with a greater content of β strand at the expense of pPII as reflected in the Gaussian model (Figure 5). It also has the most complex Ramachandran distributions, characterized by the presence of pβ instead of βt and aβ basins as well as type I/II′ β-turn and left-handed helical basins, which are mostly absent from the conformational ensembles of other aliphatic guest residues. For isoleucine in GIG, the Ramachandran distribution of Amber ff24EXP-GA exhibits type I/II′ β-turn and left-handed helical turn supporting conformations, in contrast to Ramachandran distributions of Amber ff14SB and CHARMM36m. Of the three MD force fields, Amber ff24EXP-GA produces the most complex Ramachandran distributions for I (Figure 5). These qualitative observations are supported by a quantitative comparison to spectroscopic data in Figure S14, which demonstrates that Amber ff24EXP-GA produces the lowest χ_*J*_^2^, χ_*J*ϕ_^2^, and χ_VCD_^2^ values among the three force fields.

#### Aromatic Amino Acid Residues: F and Y

**Figure 6:**
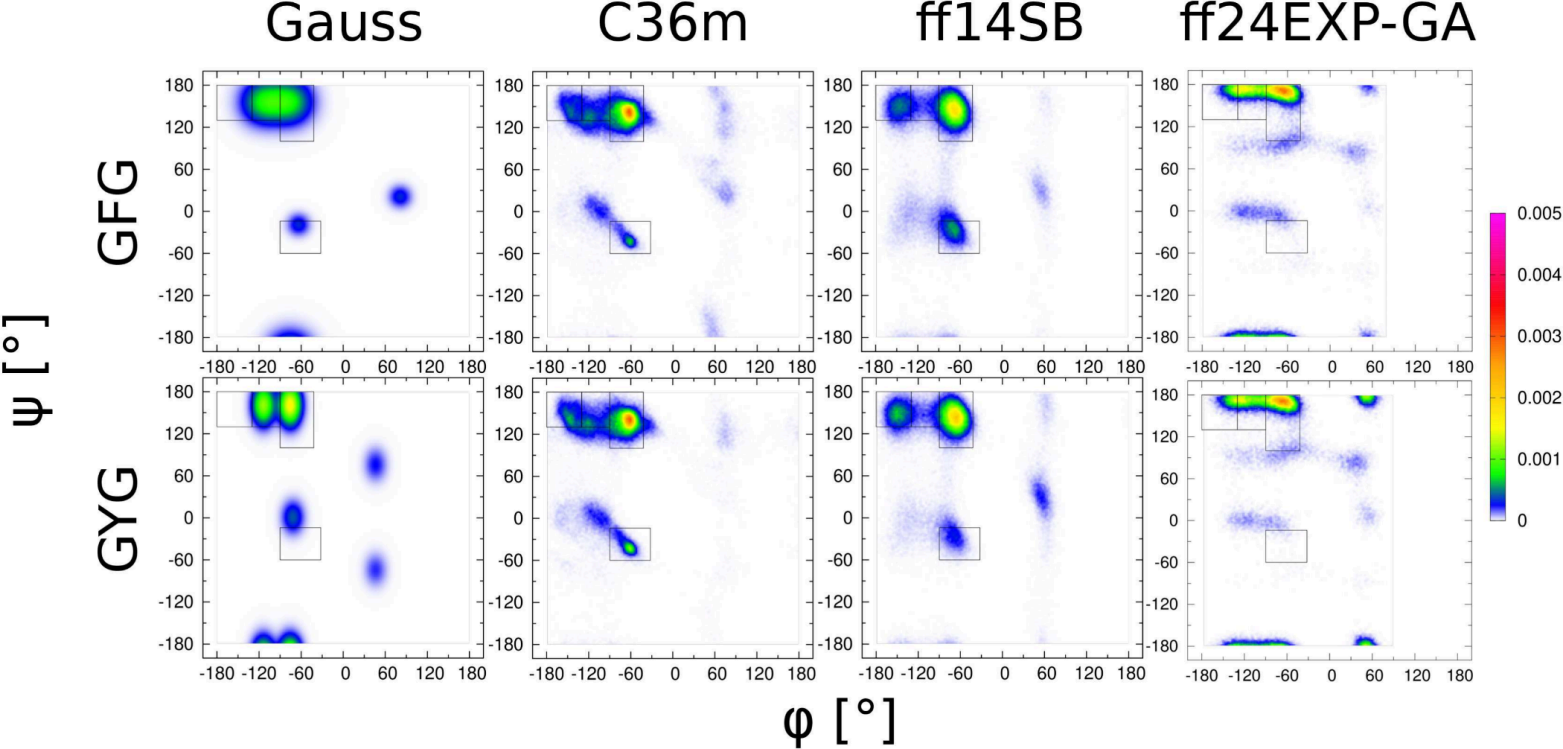
Ramachandran distributions of aromatic guest amino acid residues GxG, x = F, Y, for different MD force fields assessed and the Gaussian model.

The Gaussian model predicts somewhat distinct Ramachandran distributions for phenylalanine (F) and tyrosine (Y). In contrast, all three force fields produce similar Ramachandran distributions (Figure 6). In Amber ff24EXP-GA, the same backbone dihedral potential (glycine-like) is used for both of these residues. In all three MD force fields, the OH group in the phenyl ring in tyrosine in GYG does seem to significantly affect the conformational ensembles, resulting in similar Ramachandran distributions for phenylalanine and tyrosine. Nonetheless, the Amber ff24EXP-GA force field produces the lowest χ_*J*_^2^ and χ_*J*ϕ_^2^ values among the three MD force fields for both F and Y residues (compare Figures S15(vii,viii) and S16(vii,viii)). These residues have comparable *J*-coupling constants and only differ from each other in terms of the amide I′ VCD profiles. With respect to VCD amide I′ profiles, the χ_VCD_^2^ for phenylalanine is the lowest for Amber ff14SB, followed by Amber ff24EXP-GA and CHARMM36m (Figure S14(ix)). For tyrosine, Amber ff24EXP-GA produces the highest χ_VCD_^2^ value among the three force fields (Figure S15(ix)).

#### Polar Nonionizable Amino Acid Residues: C, N, S, and T

**Figure 7:**
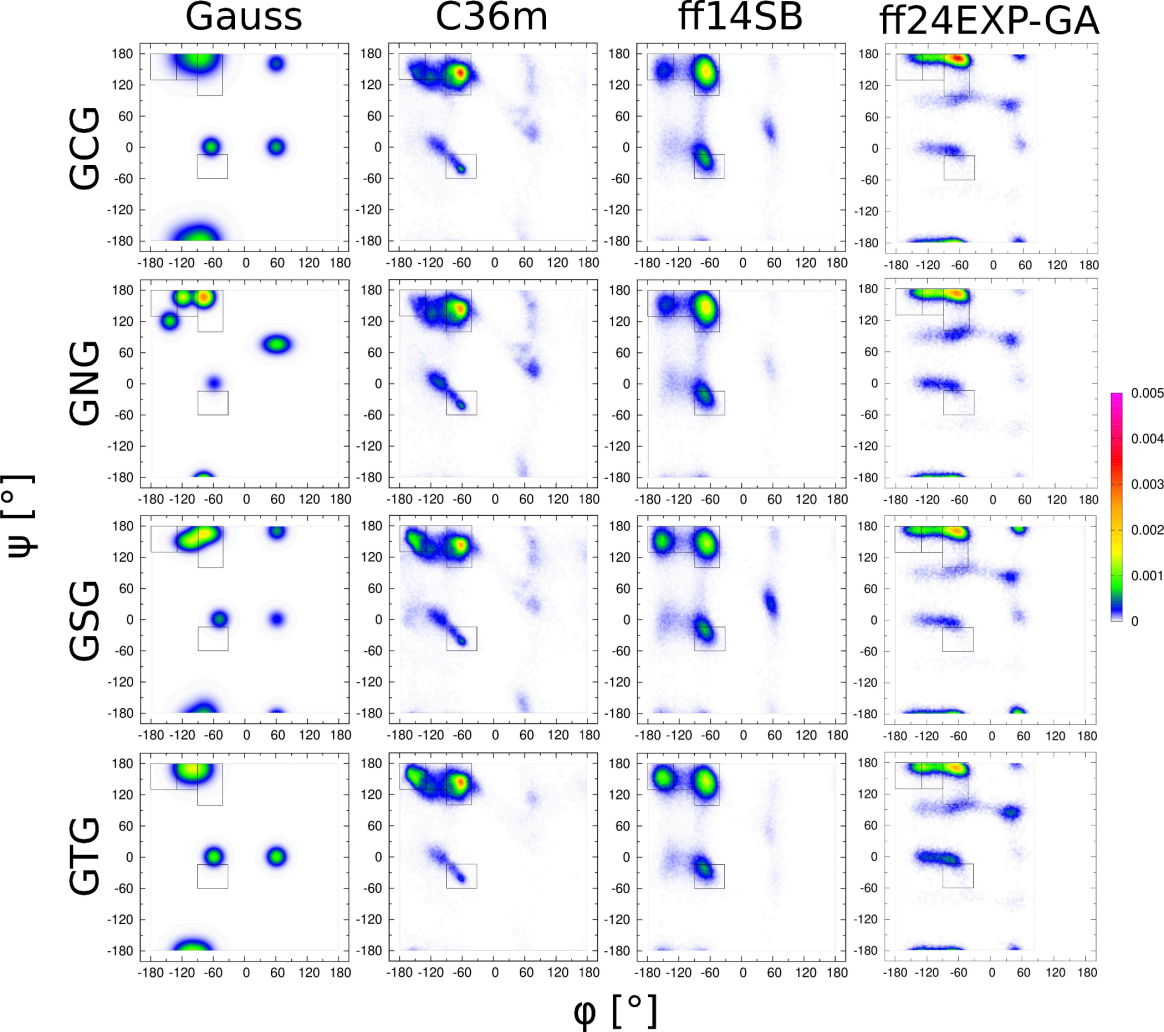
Ramachandran distributions of polar guest amino acid residues GxG, x = C, N, S, T, for different MD force fields assessed and the Gaussian model.

These amino acid residues have more chemically diverse side chains that vary widely in terms of polarity, hydrogen bonding capability, and the chemical nature of the respective functional groups. As expected, the Gaussian Ramachandran distributions of these four guest residues show a high degree of variability. All three MD force fields resulted in similar Ramachandran distributions for C and N. A similar observation can be made for the Ramachandran distributions of the S and T. MD-derived Ramachandran distributions for C and N are visibly different from those for S and T (See Figure 7). Nonetheless, these distributions are strongly force-field-specific. Among the three force fields, Amber ff24EXP-GA exhibits the least variability in the Ramachandran distribution across the residues in this group. The Gaussian Ramachandran distribution of guest cysteine in GCG is characterized by significant populations of the type I/II′ β_*i*+2_ turn-like, type I′/II β_*i*+2_ turn-like, and asx turn-like regions (70 < ϕ < 110, 75 < ψ < 170). Amber ff24EXP-GA exhibits a better sampling of the aforementioned turn regions than the other two force fields, resulting in the lowest χ_*J*_^2^ value and a χ_*J*ϕ_^2^ value comparable to CHARMM36m (Figure S17vii–ix). It is worth noting that of the three MD force fields, Amber ff24EXP-GA is the only one that predicts a nonzero population in the asx-turn region. The guest asparagine in GNG is characterized by a complex Gaussian Ramachandran distribution with significant populations in the pPII, type I/II′ β_*i*+2_ turn, extended β, and left handed helical regions (Figure 7). The extended β region is not populated in any of the three MD force fields. The Ramachandran distribution derived within Amber ff24EXP-GA shows significant occupancy in these regions (other than the extended β region) as opposed to the other two force fields. All three MD force fields predict relatively high χ_*J*_^2^, χ_*J*ϕ_^2^, and χ_VCD_^2^ values. CHARMM36m results in the lowest χ_*J*_^2^ and χ_*J*ϕ_^2^ values, followed by Amber ff24EXP-GA and Amber ff14SB (Figure S18(vii,viii)). In the case of the amide I′ profile, χ_VCD_^2^ is lowest for Amber ff14SB, followed by Amber 24EXP-GA and CHARMM36m with comparable values (Figure S18(vi,ix)). The Gaussian Ramachandran distributions of guest serine and threonine in GSG and GTG, respectively, are similar, except for the nonzero population of the asx-turn region in the case of serine (which is not present in the case of threonine). In the case of serine, only Amber ff24EXP-GA does not overestimate the aβ mesostate at the expense of βt mesostate (Figure 7), while also showcasing a larger population of the type I/II′ β_*i*+2_, type I′/II β_*i*+2_, and asx turns, consistent with the Gaussian Ramachandran distribution. Amber ff24EXP-GA shows the lowest χ_*J*_^2^ and the second lowest χ_*J*ϕ_^2^ following and comparable to CHARMM36m (Figure S19(vii,viii)). Amber ff24EXP-GA results in the highest albeit comparable χ_VCD_^2^ value among the three force fields (Figure S19(ix)). In the case of guest threonine, of the three force fields, Amber ff24EXP-GA produces the most significant βt population and an increased sampling in the region with ϕ < 0, resulting in the lowest χ_*J*_^2^, χ_*J*ϕ_^2^, and χ_VCD_^2^ values among the three MD force fields (Figure S20(vi–ix)).

#### Polar Ionizable Amino Acid Residues: D^p^, E^p^, and R

**Figure 8:**
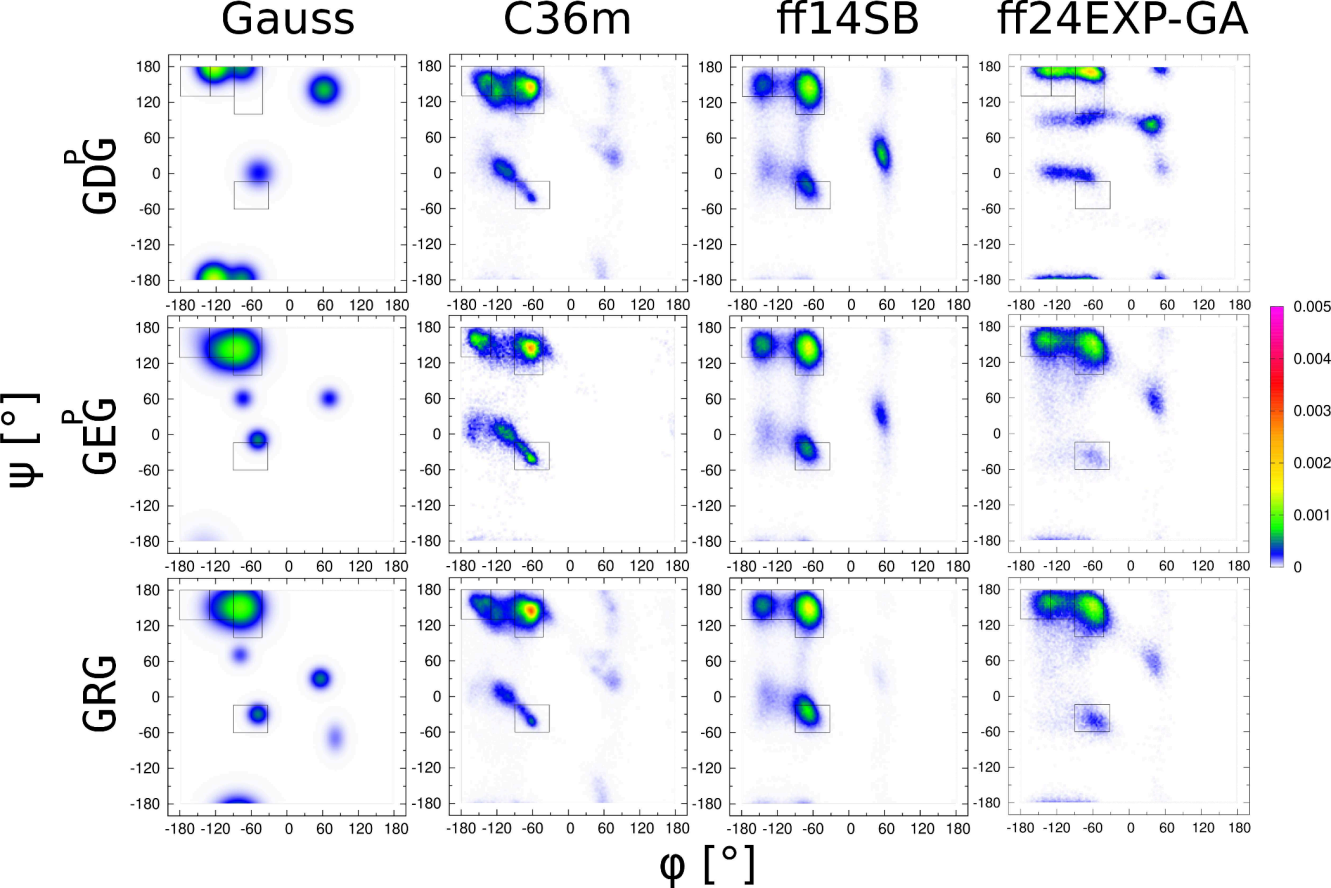
Ramachandran distributions of charged guest amino acid residues GxG, x = D^p^, E^p^, R, for different MD force fields assessed and the Gaussian model.

The residues in this group are characterized by functional groups that are polar and contain one ionizable hydrogen atom. Guest protonated aspartic acid and glutamic acid residues have a carboxylic acid group (−COOH) at the β- and γ-carbon atoms, respectively. The side chain carboxylic acid groups are protonated to mimic the experimental conditions. Guest arginine has an amphipathic side chain with a guanidino group attached to the δ-carbon. The guanidino group is protonated at physiological and low pH. All these residues have high propensities for hydrogen bond formation, which can lead to the side chain interacting with the backbone amide groups to influence the conformational dynamics of the peptide backbone. For the guest protonated aspartic acid residue in GD^p^G, the Gaussian model shows an increased population in the βt region at the expense of the pPII region. In addition, a protonated aspartic acid also favors type I/II′ β_*i*+2_ and asx turns. Of the three force fields, Amber ff24EXP-GA produces the highest asx turn population (Figure 8), which is reflected in the lowest χ_*J*_^2^ and χ_*J*ϕ_^2^ values among the three force fields (Figure S21(vii,viii)). The χ_VCD_^2^ value predicted by Amber ff24EXP-GA is comparable to that of the Gaussian model. The lower χ_VCD_^2^ values predicted by Amber ff14SB and CHARMM36m can be attributed to the overestimation of pPII, which compensates for the underestimated asx turn population (Figure S21(ix)). The polar functional group of GD^p^G is in closer proximity to the backbone than the polar functional groups of GE^p^G and GRG; hence the Ramachandran distribution of guest D^p^ in GD^p^G is significantly different from that of guests E^p^ and R. Among the three force fields, Amber ff24EXP-GA produces similar Ramachandran distributions for E^p^ and R that differ from that of D^p^ (Figure 8). For the D^p^ residue in cationic GD^p^G, the Amber ff24EXP-GA force field has the lowest χ_*J*_^2^ followed by CHARMM36m and Amber ff14SB, respectively (Figure S21(vii)). For χ_*J*ϕ_^2^ value, CHARMM36m has the lowest value, closely followed by Amber ff24EXP-GA (Figure S21(viii)). In the case of central E^p^ in cationic GE^p^G, Amber ff24EXP-GA force field has the lowest χ_*J*_^2^ value followed by Amber ff14SB and CHARMM36m force fields (Figure S22(vii)). In the case of χ_*J*ϕ_^2^, the accuracy follows the opposite order (Figure S22(viii)). In the case of R in cationic GRG, CHARMM36m exhibits the lowest χ_*J*_^2^ and χ_*J*ϕ_^2^ values, trailed by Amber ff24EXP-GA and Amber ff14SB, respectively (Figure S23(vii,viii)).

### Amber ff24EXP-GA Outperforms Amber ff14SB with Respect to Intrinsic Conformational Dynamics of 14 Guest Residues x in Cationic GxG Peptides

**Figure 9:**
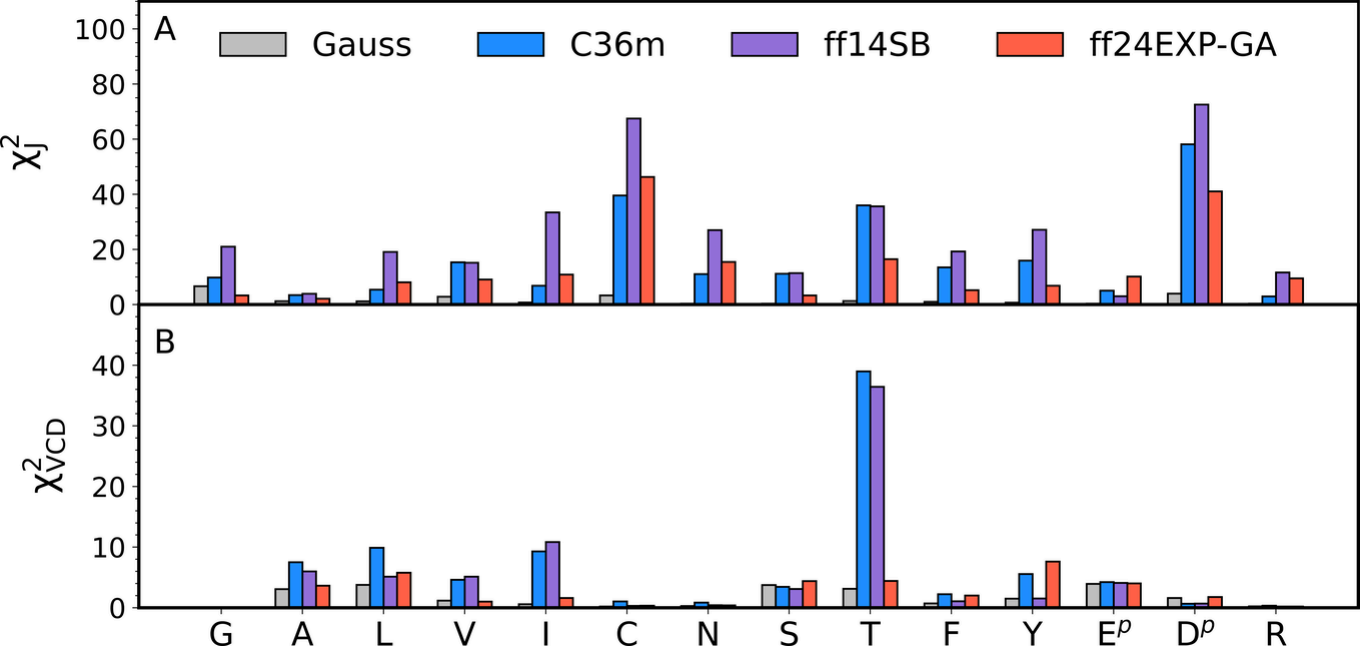
Assessment of the Gaussian model and MD force fields with respect to their ability to reproduce the experimental data for the guest residue in cationic GxG in water using (A) χ_*J*_^2^ and (B) χ_VCD_^2^ functions. Reduced χ_VCD_^2^ values for GGG are not available for the central glycine in GGG, as reported previously.^33^

Comparison of χ_*J*_^2^ values among the three MD force fields in Figure 9A demonstrates that Amber ff24EXP-GA outperforms the parent force field, Amber ff14SB, with respect to the intrinsic conformational dynamics of guest residue x in cationic GxG peptides. This is not a trivial result, because Amber ff24EXP-GA differs from Amber ff14SB only in the parametrization of the dihedral potentials, which have been simplified to only two types of dihedral potentials for backbone dihedral angles ϕ and ψ, glycine-like and alanine-like, calibrated to best account for spectroscopic data of guest glycine and alanine in GxG peptides, whereas the prime dihedral potential (depending on ϕ′ and ψ′) has been set to zero. As shown in Figure 9A, Amber ff24EXP-GA results in χ_*J*_^2^ values that are significantly lower than the corresponding Amber ff14SB values for all 14 guest residues x in cationic GxG peptides. Moreover, a comparison of Amber ff24EXP-GA to CHARMM36m in Figure 9A shows that Amber ff24EXP-GA predicts lower χ_*J*_^2^ values for 10 out of 14 guest residues, although it was not specifically calibrated for CHARMM36m. In Amber ff24EXP-GA, the largest improvement with respect to the χ_*J*_^2^ value relative to both Amber ff14SB and CHARMM36m has been made for the three residues that deviate the most from the spectroscopic data: C, T, and D^p^.

The χ_VCD_^2^ values predicted by the three MD force fields and the Gaussian model are shown in Figure 9B. With respect to the VCD amide I′ profiles, Amber ff24EXP-GA shows significant improvement over the parent ff14SB for A, V, I, and T, whereas for the other guest residues, the χ_VCD_^2^ values remain comparable to those of the parent force field or slightly increased. The exception may be Y, for which the Amber ff24EXP-GA χ_VCD_^2^ value is larger than that in the parent Amber force field and in CHARMM36m. However, the corresponding χ_*J*_^2^ value is significantly lower for Amber ff24EXP-GA than for the other two MD force fields, which balances out the less than optimal χ_VCD_^2^ value. We should nonetheless mention in this context that the amide I′ VCD of GYG is rather strange and unusual due to the presence of a positively biased negative couplet. Generally, the couplets of short peptides are either slightly negatively biased or symmetric. It is therefore not entirely clear whether the theory utilized to simulate the VCD signal is sufficient.^27^

**Figure 10:**
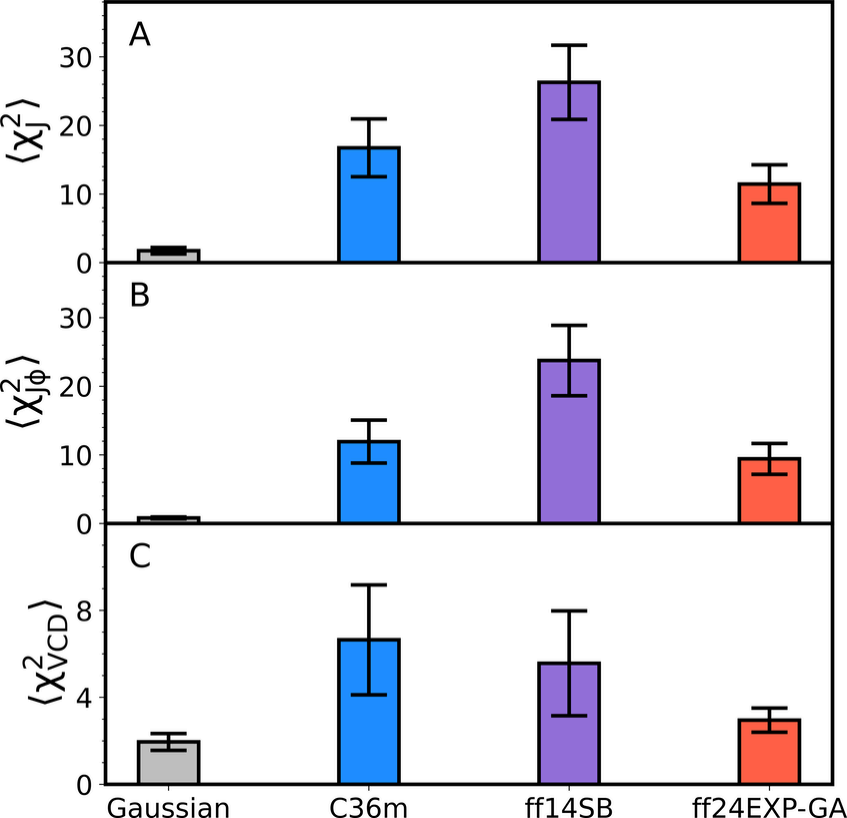
Average (A) χ_*J*_^2^, (B) χ_*J*ϕ_^2^, and (C) χ_VCD_^2^ calculated over 14 residues (G, A, L, V, I, F, Y, C, N, S, T, D^p^, E^p^, and R) for the Gaussian model and CHARMM36m, Amber ff14SB, and Amber ff24EXP-GA. The error bars correspond to the SEM values.

To further assess the level of improvement of Amber ff24EXP-GA relative to Amber ff14SB and CHARMM36m, we show ⟨χ^2^⟩ values for the Gaussian model and the three MD force fields in Figure 10, which are χ^2^ values averaged over all 14 guest amino acid residues. Notably, both ⟨χ_*J*_^2^⟩ and ⟨χ_*J*ϕ_^2^⟩ values are significantly improved in Amber ff24EXP-GA relative to its parent force field. This means that all *J*-coupling constants, not only ^1^*J*(N, C_α_) (which uniquely depends on dihedral angle ψ) are closer to their experimental values in the new force field. Interestingly, Amber ff24EXP-GA also predicts lower ⟨χ_*J*_^2^⟩ and ⟨χ_*J*ϕ_^2^⟩ values than CHARMM36m, although the error bars overlap, so the improvement over CHARMM36m may not be as significant as that over Amber ff14SB. Amber ff24EXP-GA also leads to a lower ⟨χ_VCD_^2^⟩ value than its parent force field and predicts a significantly lower ⟨χ_VCD_^2^⟩ value than CHARMM36m. With respect to intrinsic conformational dynamics of guest residues x in cationic GxG peptides, Amber ff24EXP-GA outperforms the parent force field and compares favorably to CHARMM36m. This result is impressive considering that the new modifications are rather minor and aimed to optimize only two guest residues, glycine and alanine, in GxG peptides. Despite the above improvements, the Gaussian model still outperforms Amber ff24EXP-GA by about an order of magnitude.

**Figure 11:**
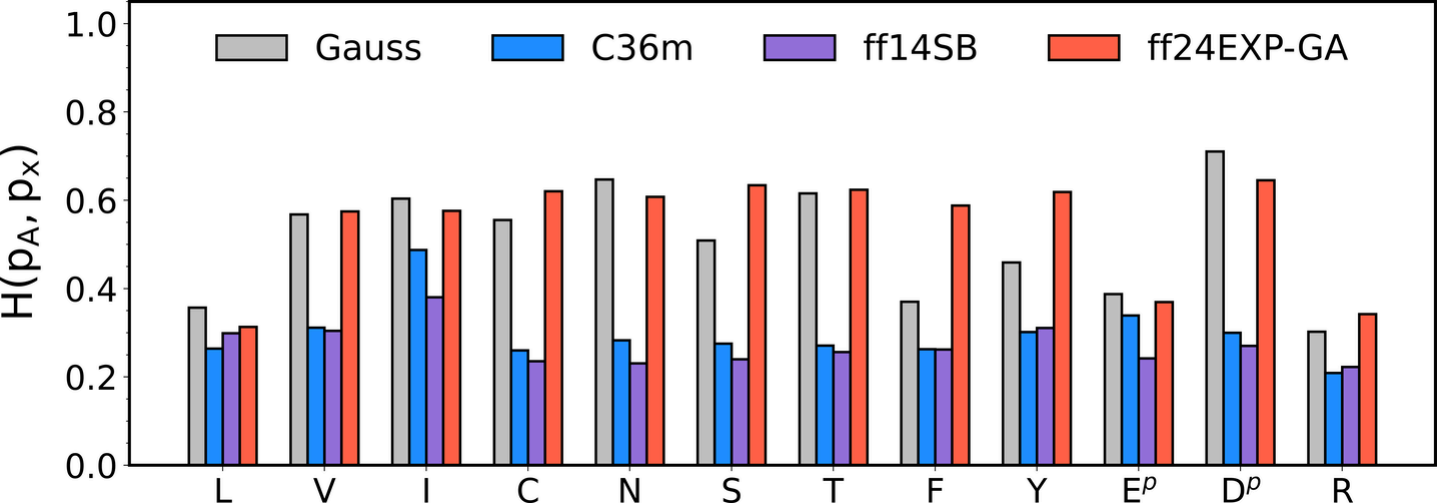
Hellinger distance between the Ramachandran distributions of residue x and alanine. This metric shows how the two Ramachandran distributions differ from one another.

In their MD force field assessment study, Andrews and collaborators reported a lack of residue specificity in intrinsic conformational ensembles of guest residues x in GxG peptides as reflected in their respective Ramachandran distributions for Amber ff14SB, CHARMM36m, and OPLS-AA/M.^22^ In contrast, the benchmark spectroscopic data-based Gaussian Ramachandran distributions exhibit considerable variability across the guest residues (Figures 5, 6, 7, 8). We asked here if Amber ff24EXP-GA offers any improvement with respect to the guest residue specificity over its parent force field Amber ff14SB or CHARMM36m. To quantify the residue specificity of the Ramachandran distributions, we calculate the Hellinger distance between the Ramachandran distribution of each guest residue x (other than glycine) and guest alanine, *H*(*p*_A_, *p*_x_) (Figure 11). Here, larger *H*(*p*_A_, *p*_x_) values indicate larger deviations (dissimilarities) from the Ramachandran distribution of guest alanine and hence a greater guest residue specificity of the force field of interest. In order to assess the significance of the obtained Hellinger distances, we adopt the criteria of Schweitzer-Stenner and Toal,^76^ which are based on the slightly less specific criteria of Ting and collaborators.^71^ If the Hellinger value lies between 0 and 0.1 distributions are considered very similar. Values in the intervals 0.1 < *H* ≤ 0.25 and 0.24 < *H* ≤ 0.4 indicate moderately similar and moderately dissimilar distributions, respectively, whereas *H*-values above 0.4 indicate very dissimilar distributions. The results in Figure 11 demonstrate that among the three force fields, Amber ff24EXP-GA exhibits overall larger *H*(*p*_A_, *p*_x_) values (*H* values) across the residues than Amber ff14SB and CHARMM36m. Large *H* values in Amber ff24EXP-GA are associated with “glycine-like” residues that share the same backbone dihedral potential parameters. Because the respective Ramachandran distributions are compared to the Ramachandran distribution of alanine in GAG, which is modeled using “alanine-like” backbone dihedral potential parameters, the resulting *H* values are large. Figure 11 demonstrates that the *H* values within Amber ff24EXP-GA are comparable to those predicted by the Gaussian model. The Gaussian model based *H*-values are mostly in the very dissimilar regime, and the remaining ones indicate that the respective distributions are moderately dissimilar from the respective alanine distribution. This tendency is qualitatively reproduced by the distributions produced by Amber ff24EXP-GA. In contrast, distributions obtained with Amber ff14SB and CHARMM36m are associated with *H*-values mostly at the boundary between moderately similar and moderately dissimilar distributions, consistent with the lack of amino acid residue specificity that was previously reported.^22^ Amino acid specificity of Amber ff24EXP-GA relative to the other two MD force fields is reflected also in the respective mesostate populations and free energy estimates, which are displayed in Tables S4 and S5. Whereas χ_*J*_^2^ and χ_VCD_^2^ values are based on the entire Ramachandran distributions and thus are independent of the definition of mesostates, it is important to note that mesostate populations measure the occupancies of specific regions of the Ramachandran space (as defined in Methods), which may not overlap with the entire conformational ensembles. For example, the pPII basin of the guest glycine residue in GGG is shifted to higher ψ values relative to the pPII basin of the guest alanine residue in GAG, and, consequently, a considerable pPII population falls out of the pPII mesostate region.^33^ Likewise, free energy estimates in Tables S4 and S5, which are obtained as averages of the potential of the mean force within each mesostate relative to unoccupied regions of the Ramachandran space (which are associated with zero free energy), depend on the definition of the mesostates. Thus, mesostate populations and free energy estimates in Tables S4 and S5 offer only a rough comparison among the force fields and the Gaussian model.

One of the key findings of this present study is that reproducing the intrinsic conformational ensembles of guest glycine in GGG significantly affects the ability of the MD force field to reproduce the intrinsic conformational ensembles of other amino acid residues in water. This is not a surprise, considering that GGG can be viewed as a model of a peptide backbone and that hydrogen bonding between water and the peptide backbone stabilizes the prevalent pPII state shared across most residues.^33^ Several MD force fields aiming to reproduce experimental data for IDPs have emerged in the past decade. Andrews and collaborators have assessed CHARMM36m, for example, which was shown to reproduce the intrinsic conformational ensembles of guest glycine in GGG in water quite well^22,33^ although not as well as Amber ff24EXP-GA. We here asked how well Amber ff03ws (with TIP4P-2005) with modified short-range peptide–water interactions,^10^ Amber 99SB-Disp (with TIP4P-D water) that was designed for both folded and disordered proteins,^14^ or protein coil library-based CHARMM36IDPSFF (with TIP3P) with residue-specific dihedral potentials^18^ capture the intrinsic conformational ensembles of guest glycine in GGG. Figure S24 shows the comparison of these three force fields, Amber ff24EXP-GA, and the Gaussian model to five *J*-coupling constants, of which only ^1^*J*(N, C_α_) depends on ψ and the other four constants depend on ϕ (amide I′ profiles are not shown as the they are vanishingly small for this achiral system). Overall, Amber ff24EXP-GA outperforms the other three MD force fields with respect to reduced χ_*J*_^2^ and χ_*J*ϕ_^2^ values as well as with respect to individual *J*-coupling constants, except for ^3^*J*(H^N^, H^C_α_^), which is slightly better reproduced in Amber 99SB-Disp than in Amber ff24EXP-GA. These results demonstrate that an improvement in peptide–water interactions alone and the protein coil library-based approach do not lead to a better reproduction of intrinsic conformational propensities of amino acid residues in water.

### Performance of Amber ff24EXP-GA on Longer Peptides

We here briefly assess the performance of Amber ff24EXP-GA on six longer peptides/proteins and compare the results to those obtained within CHARMM36m and Amber ff14SB. As described in Methods, nine 1 μs long MD trajectories were acquired for each of the six proteins, i.e., three replica MD trajectories for each of the three force fields.

**Figure 12:**
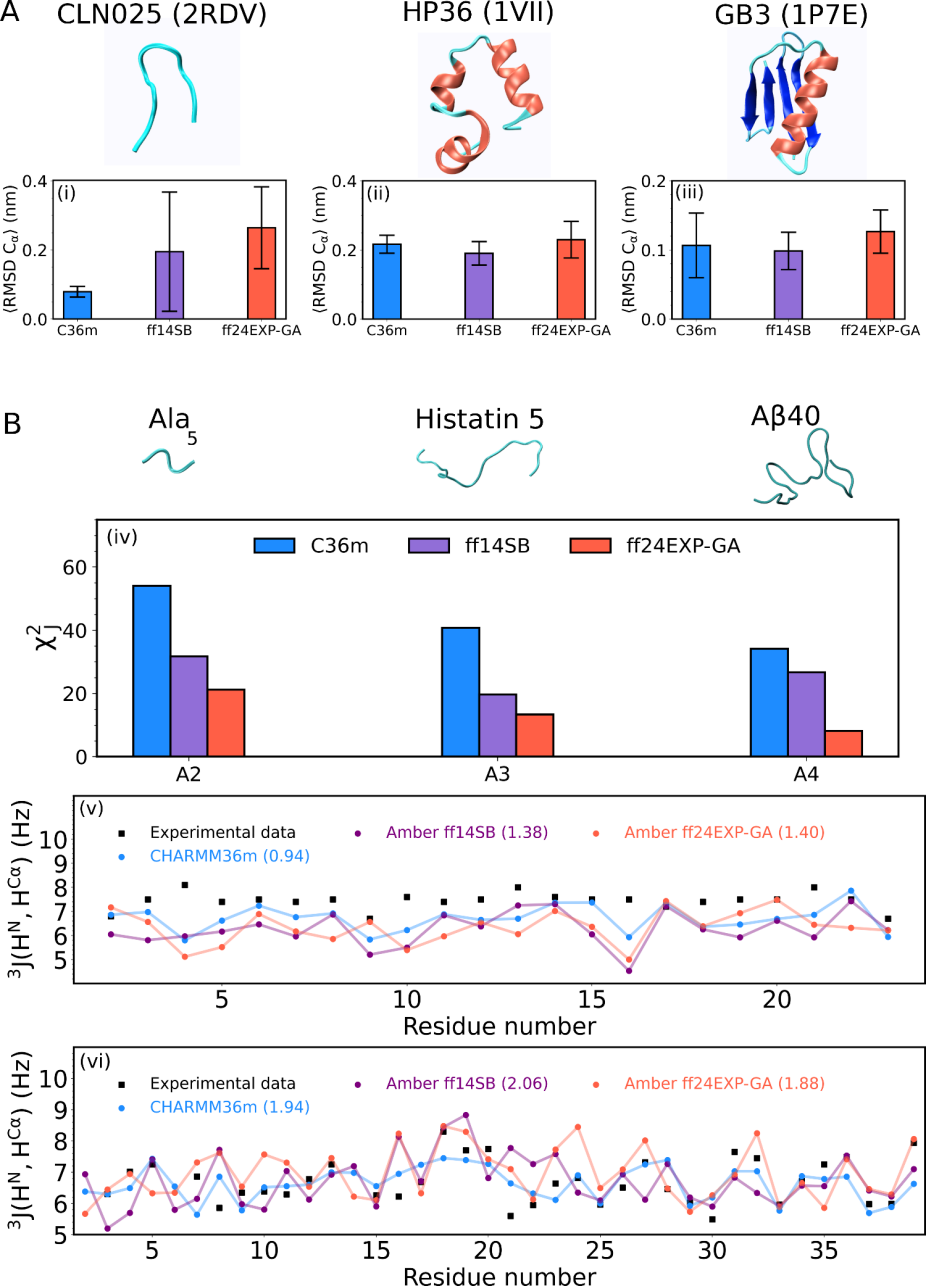
(A) Three proteins with experimentally resolved folds examined for stability within the three MD force fields by calculating the average RMSD values and standard deviations for (i) CLN025 (PDB-ID: 2RDV(56)), (ii) HP36 (PDB-ID: 1VII(57)), and (iii) GB3 (PDB-ID: 1P7E(58)). (B) Three IDP peptides examined by the three MD force fields and compared to available spectroscopic data. (iv) Reduced χ_*J*_^2^ values for residues A2, A3, and A4 of Ala_5_ calculated from the experimental^59^ and MD *J*-coupling constants shown in Figure S25 and tabulated in Table S6. Experimental^60,61^ and MD-derived per-residue ^3^*J*(H^N^,H^C_α_^) values for (v) histatin 5 and (vi) Aβ40.

Three proteins with experimentally resolved structures, (i) chingnolin (CLN025), (ii) villin headpiece (H36), and (iii) third immunoglobulin binding domain of protein G (GB3), were selected to examine and compare the stability of the three native folds within CHARMM36m, Amber ff14SB, and Amber ff24EXP-GA. The average C_α_ atom-based RMSD values and respective standard deviations in Figure 12A, which provide a measure of deviations from the experimental structures, demonstrate that all three force fields capture the native folds of these proteins fairly well. Notably, Amber ff24EXP-GA exhibits the largest fluctuations around the native fold of CLN025, suggesting that this fold is somewhat less stable in Amber ff24EXP-GA than in the other two force fields. CHARMM36m predicts the lowest RMSD values, consistent with the most stable CLN025 fold. The average coil, turn, strand, and α-helical content in these three proteins displayed in Figure S25 demonstrate that the three MD force fields produce comparable helix and helix/strand content of H36 and GB3, respectively, whereas, of the three force fields, only CHARMM36m produces significant strand content of CLN025, which contributes to the stability of the fold within this force field.

In addition to the proteins with resolved structures, we also selected three IDPs: (iv) pentaalanine (Ala_5_), (v) histatin 5, and (vi) amyloid β-protein 1–40 (Aβ40), all of which lack unique native folds. IDPs are challenging to study, in part due to limited spectroscopic data available for comparison to theoretical results. Ala_5_ was chosen because experimental values of multiple *J*-coupling constants for A2, A3, and A4 in this peptide have been reported.^59^ The comparison between experimental and MD-derived values of five *J*-coupling constants for A2 and A4 and four *J*-coupling constants for A3 (Table S6) is displayed in Figure S26 and the respective reduced χ_*J*_^2^ values are shown in Figure 12B(iv). The comparison of χ_*J*_^2^ values demonstrates that Amber ff24EXP-GA outperforms both CHARMM36m and Amber ff14SB with respect to the reported *J*-coupling constants, indicating that the dihedral potential optimization for the alanine residue in GAG in Amber ff24EXP-GA propagates to longer alanine-based sequences. For histatin 5 and Aβ40, experimental values of ^3^*J*(H^N^, H^C_α_^) have been reported for most residues along the two sequences by Raj et al.^60^ and Roche et al.,^61^ respectively. Experimental and MD-derived ^3^*J*(H^N^, H^C_α_^) values for histatin 5 and Aβ40 are displayed in Figure 12B(v,vi), respectively. The RMS deviations from the experimental per-residue ^3^*J*(H^N^, H^C_α_^) values, averaged over all residues, for histatin 5 are comparable among the three MD force fields, amounting to 0.94, 1.38, and 1.40 Hz obtained within CHARMM36m, Amber ff14SB, and Amber ff24EXP-GA, respectively. It should be noted that the experimental ^3^*J*(H^N^, H^C_α_^) values are in the range between 7 and 8 HZ and do not vary considerably along the histatin 5 sequence, which may be indicative of balanced pPII/β-strand populations. The respective MD-derived *J*-coupling constants are systematically lower than the respective experimental values at residues A4, G9 or Y10, and E16, indicating increased sampling of pPII and/or helical populations. Amber ff24EXP-GA performs somewhat worse than its parent force field, while CHARMM36m outperforms both Amber force fields. This result is not overly surprising considering that the amino acid sequence of histatin 5 is dominated by histidine, which has not been included in the optimization of Amber ff24EXP-GA. In analogy, the average RMS deviations from the experimental per-residue ^3^*J*(H^N^, H^C_α_^) values for Aβ40 are comparable for the three MD force fields, amounting to 1.94, 2.06, and 1.88 Hz derived within CHARMM36m, Amber ff14SB, and Amber ff24EXP-GA, respectively. In this case, Amber ff24EXP-GA performs somewhat better than the other two force fields. The above findings provide evidence that Amber ff24EXP-GA performs well on longer peptides and proteins while outperforming parent Amber ff14SB on short unfolded peptides.

## Conclusions and Discussion

MD is a powerful tool for elucidating conformational dynamics of IDPs, which is often critical for understanding their multifaceted biological functions at a level of detail not accessible to experiments. In contrast to globular proteins, IDPs are characterized by amino acid sequences that are exposed to the solvent and adopt multiple distinct conformations depending on their local environment and/or binding partners. The lack of a unique native fold of an IDP combined with an increased exposure of IDP’s sequence to the solvent poses many challenges to both experimental and computational studies that aim to characterize its conformational ensembles. We here posit that any MD force field suited for studies of IDPs needs to be able to reproduce the intrinsic conformational dynamics of amino acid residues in water, in line with experimental constraints. To this end, we focus on GxG peptides with a guest residue x, flanked by two glycines to minimize the neighboring effects, as model peptides that probe intrinsic conformational dynamics of guest amino acid residue x in water. A comprehensive set of spectroscopic data, including 5 *J*-coupling constants and VCD amide I′ profiles, for each of 14 guest residues x in cationic GxG peptides in water has been acquired by Schweitzer-Stenner and collaborators over the past 15 years.^25−29^ Importantly, each set of spectroscopic data sufficiently constrains the Ramachandran space of backbone dihedral angles ϕ and ψ to allow for a meaningful application of the Gaussian superposition model, which can be used derive experiment-based guest-residue specific Ramachandran distribution of guest residue x as a sum of 2D Gaussian subdistributions associated with various mesostates, whereby the parameters are adjusted to obtain the best fit to the spectroscopic data.^30^ These experimentally based Gaussian Ramachandran distributions can be used as benchmark distributions for evaluation of MD force fields with respect to their ability to capture intrinsic conformational dynamics of amino acid residues in water. Despite notable advances in the development of MD force fields in the past decade, recent assessment studies that probed their capacity to reproduce intrinsic conformational dynamics of guest amino acid residues x in GxG peptides in water have revealed a less than ideal agreement with the available spectroscopic data.^22,32,33,44^ Using reduced χ_*J*_^2^ and χ_VCD_^2^ values as assessment measures for several MD force fields (Amber ff14SB, Amber ff19SB, OPLS-AA/M, and CHARMM36m), Andrews and collaborators reported that the Gaussian Ramachandran distributions for 14 guest residues x overall outperform MD-derived Ramachandran distributions by at least one order or magnitude.^22^ The most notable weaknesses in MD-derived Ramachandran distributions of guest residues x in GxG peptides have been (i) the lack of guest residue specificity, including insufficient variability of the pPII population among the guest residues, (ii) oversampling of the aβ at the expense of the βt region, and (iii) inadequate sampling of turn-forming conformations for ionizable and polar residues.^22^

The lack of amino acid residue specificity in conformational ensembles, as reflected in Ramachandran distributions derived from MD force fields, has been tackled by several groups. Wu and collaborators utilized protein coil libraries to derive mean-field type Ramachandran distributions for individual amino acid residues, which served as target distributions in the IBI method, similar to the method used in this work, aimed to optimize OPLS-AA/L^77,78^ and Amber ff99SB^79^ force fields and obtain residue-specific MD force fields, RSFF1^20^ and RSFF2,^21^ respectively. Because protein coil libraries contain a large amount of experimental data from numerous X-ray diffraction and solid-state NMR studies, the resulting Ramachandran distributions are robust; however, they represent averages over all possible neighboring amino acid residues and do not necessarily reflect the intrinsic conformational propensities of amino acid residues in water. This is reflected in the oversampling of the aβ at the expense of the βt region, for example, and leads to less than ideal reproduction of spectroscopic data of intrinsic conformational ensembles.^22^ In CHARMM36m, backbone dihedral potentials are implemented as two-dimensional CMAPs, which introduces more degrees of freedom into the potentials. Nonetheless, Jiang and collaborators recently reported a generalized IBI method that can be used to optimize residue-specific CMAP potentials.^80^ Another approach was taken by Simmerling and collaborators, who implemented residue-specific CMAP potentials into Amber ff14SB using target data derived from quantum-mechanical implicit-solvent calculations of dihedral potentials for all dipeptides, resulting in Amber ff19SB.^9^ An assessment of Amber ff19SB with respect to intrinsic conformational dynamics of guest residue x in GxG peptides, although promising with respect to capturing residue specificity, exhibits large deviations from spectroscopic data.^22^

In this work, we introduce a new Amber force field obtained by optimization of the backbone dihedral potential function of the parent Amber ff14SB force field. In our minimalistic approach, we hypothesized that improving the backbone dihedral potentials for guest glycine and alanine residues in GxG peptides alone will improve the ability of the force field to reproduce the spectroscopic data for all guest amino acid residues in water. To understand the optimization procedure, it is important to consider that the potential energy in the parent Amber ff14SB force field includes two types of backbone dihedral potentials, (i) non-prime potential *V*(ϕ, ψ), which is nonzero for all amino acid residues and does not depend on the position of the side-chain C_β_ atom, and (ii) *V*(ϕ′, ψ′), which is nonzero for all residues other than glycine and depends on the position of the side chain C_β_ atom. In our approach, we set the prime potential to zero, *V*(ϕ′, ψ′) = 0, and focus exclusively on optimization of non-prime dihedral angle potential *V*(ϕ, ψ), whereby our target systems are guest glycine and alanine residues in cationic GGG and GAG, respectively. Using nonchiral GGG as a means to optimize the MD force field is rather novel as most MD force fields have utilized quantum-mechanical calculations and empirical experimental data-based adjustments on alanine-based short peptides to determine the dihedral potential parameters. The rationale for using intrinsic conformational ensembles for glycine is that it represents a model of a peptide backbone shared across all amino acid residues.^33^ Our optimization of dihedral potential *V*(ϕ, ψ) for glycine and alanine residues in GGG and GAG, respectively, reveals two sets of dihedral parameters, which we then apply individually to simulate the remaining 12 GxG peptides so as to determine which of the two types of parameters (glycine-like or alanine-like) is better suited to capture the spectroscopic data for each specific guest residue x. Interestingly, 9 out of the remaining 12 guest residues are better described using glycine-like dihedral potential parameters, showcasing the importance of correctly capturing the conformational dynamics of glycine and, by implication, the peptide backbone. In the new MD force field, which we refer to as Amber ff24EXP-GA, 12 of the remaining amino acid residues are classified as glycine-like or alanine-like, depending on the optimal set of backbone dihedral parameters, and the remaining amino acid residues are classified into these two categories *ad hoc* by considering side chain similarities of the residue of interest to glycine-like or alanine-like residues. Because all simulations of cationic GxG peptides in water within Amber ff14SB and other Amber force field variants reported here, which are integral parts of the optimization procedure, have been acquired using the TIP4P-2005 water model, we recommend that the new force field Amber ff24EXP-GA is also used in combination with this water model.

We assessed Amber ff24EXP-GA with respect to all 14 guest residues in GxG peptides, for which the complete set of spectroscopic data is available, using reduced χ_*J*_^2^ and χ_VCD_^2^ values, and compared its performance with the parent Amber ff14SB as well as CHARMM36m. Although the optimization of the backbone dihedral parameters is applied to only guest glycine and alanine residues in GGG and GAG peptides, respectively, Amber ff24EXP-GA outperforms Amber ff14SB with respect to χ_*J*_^2^ values for all 14 guest residues x in GxG peptides, for which the complete set of spectroscopic data is available. Amber ff24EXP-GA also outperforms the parent force field with respect to χ_VCD_^2^ values for 7 guest residues. A notable exception is tyrosine with the χ_VCD_^2^ value being significantly larger than the respective values for Amber ff14SB, CHARMM36m, and the Gaussian model. Although the Amber ff24EXP-GA χ_VCD_^2^ values for leucine, phenylalanine, serine, and protonated aspartic acid are slightly larger than those derived in Amber ff14SB, CHARMM36m, and the Gaussian model, they are quite comparable. Overall, Amber ff24EXP-GA results in about 2-fold reduction of the average χ_*J*_^2^ and χ_VCD_^2^ values relative to parent Amber ff14SB, if we consider the average over all 14 guest residues. Moreover, Amber ff24EXP-GA outperforms CHARMM36m with respect to reproduction of spectroscopic data for 14 guest residues in GxG peptides, whereby the average χ_*J*_^2^ and χ_VCD_^2^ values are reduced by factors of 1.5 and 2.2, respectively. This is an unexpected and powerful result, indicating that the backbone dihedral potentials in MD force fields play a critical role in modeling the conformational dynamics of amino acid residues and further showcasing the importance of the peptide backbone description in capturing the intrinsic conformational dynamics of amino acid residues in water in line with spectroscopic data. The improved performance of Amber ff24EXP-GA over Amber ff14SB and CHARMM36m on short GxG-type peptides described in this work does not guarantee that this improved description will propagate to longer peptides and proteins. A brief evaluation of CHARMM36m, Amber ff14SB, and Amber ff24EXP-GA on six longer peptides provides evidence that Amber ff24EXP-GA performs comparably to the other two force fields, while outperforming Amber ff14SB on short unfolded peptides.

## Supporting Information Available

Karplus parameters used to calculate the *J* coupling constants, backbone dihedral potential parameters, list of amino acid residues and their corresponding dihedral parameter set, mesostate populations and free energy estimates, *J*-coupling constants and uncertainties for A2–A4 of Ala_5_, plots of the Karplus curves, application of the IBI method to guest glycine and alanine in GxG peptides to 4 and 5 dihedral potential terms, convergence of dihedral coefficients, optimization procedure for GGG and GAG, plots of dihedral potentials, Ramachandran plots of central residues of all GxG peptides, comparison of χ_*J*_^2^, χ_*J*ϕ_^2^ and χ_VCD_^2^ for all amino acid residues and parameter sets, comparison of experimental and computed *J* coupling constants and amide I′ profiles of all guest residues (PDF), assessment of three MD force fields on the conformational ensemble of guest glycine residue in GGG, coil, strand, turn, and helix content in CLN025, HP36, and GB3, absolute differences between experimental and calculated *J*-coupling constants for A2–A4 in Ala_5_ (PDF)
Amber ff24EXP-GA parameters formatted for GROMACS (ZIP)
