# Supplementary material for: Amber ff24EXP-GA, Based on Empirical Ramachandran Distributions of Glycine and Alanine Residues in Water
Source: J Chem Theory Comput. 2025 Feb 20;21(5):2515–34. doi: 10.1021/acs.jctc.4c01450 (PMC11912210; doi:10.1021/acs.jctc.4c01450)
Supplement: Supplementary file 1 — ct4c01450_si_001.pdf [file ct4c01450_si_001.pdf]

Supporting Information: Amber ff24EXP-GA, Based on Empirical Ramachandran Distributions of Glycine and Alanine Residues in Water†

Athul Suresh,† Reinhard Schweitzer-Stenner,‡ and Brigita Urbanc*,†

†*Department of Physics, Drexel University, Philadelphia, PA 19104, USA*

‡*Department of Chemistry, Drexel University, Philadelphia, PA 19104, USA*

E-mail: bu25@drexel.edu

Supporting Tables

Table S1: The Karplus parameters for each of the J-coupling constants used in our analysis. The citations refer to the papers, where the respective Karplus parameters have been reported. The corresponding statistical uncertainties (unavailable for $^3J(C,C')$ and $^1J(N,C_\alpha)$) are in parentheses.

J-coupling constant [Hz]	Dihedral angle	θ_0 [°]	A [Hz]	B [Hz]	C [Hz]
$^3J(H^N,H^{C\alpha})$ [S1]	ϕ	-60	7.09 (0.11)	-1.42 (0.04)	1.55 (0.09)
$^3J(H^N,C')$ [S2]	ϕ	180	4.29 (0.14)	-1.01 (0.06)	0.00 (0.02)
$^3J(H^{C\alpha},C')$ [S2]	ϕ	120	3.72 (0.07)	-2.18 (0.08)	1.28 (0.04)
$^3J(C,C')$ [S2]	ϕ	0	1.36	-0.93	0.60
$^3J(H^N,C_\beta)$ [S2]	ϕ	60	3.06 (0.07)	-0.74 (0.04)	0.13 (0.03)
$^1J(N,C_\alpha)$ [S3]	ψ	0	1.70	-0.98	9.51

Table S2: Parameters of the backbone dihedral potential $V(\phi,\psi)$ comprising dihedral force constants $k_{n\phi}$, $k_{m\psi}$ and the phase shifts $\delta_{n\phi}$, $\gamma_{m\psi}$, as implemented in Amber ff24EXP-GA.

AA Type	Parameters							
	$k_{n\phi}$ [kJmol^{-1}]		$\delta_{n\phi}$ [°]		$k_{m\psi}$ [kJmol^{-1}]		$\gamma_{m\psi}$ [°]	
	$k_{1\phi}$	-4.74732	$\delta_{1\phi}$	0.0	$k_{1\psi}$	1.33356	$\gamma_{1\psi}$	180
	$k_{2\phi}$	-0.32510	$\delta_{2\phi}$	0.0	$k_{2\psi}$	6.40920	$\gamma_{2\psi}$	180
Gly	$k_{3\phi}$	-1.35146	$\delta_{3\phi}$	0.0	$k_{3\psi}$	0.72013	$\gamma_{3\psi}$	180
	$k_{4\phi}$	0.62734	$\delta_{4\phi}$	0.0	$k_{4\psi}$	-7.57888	$\gamma_{4\psi}$	180
	$k_{5\phi}$	0.00000	$\delta_{5\phi}$	0.0	$k_{5\psi}$	0.00000	$\gamma_{5\psi}$	0.0
	$k_{1\phi}$	-3.86525	$\delta_{1\phi}$	0.0	$k_{1\psi}$	0.29784	$\gamma_{1\psi}$	180
	$k_{2\phi}$	-1.24129	$\delta_{2\phi}$	0.0	$k_{2\psi}$	4.34385	$\gamma_{2\psi}$	180
Ala	$k_{3\phi}$	-1.16794	$\delta_{3\phi}$	0.0	$k_{3\psi}$	0.65569	$\gamma_{3\psi}$	180
	$k_{4\phi}$	-0.25300	$\delta_{4\phi}$	0.0	$k_{4\psi}$	-0.25079	$\gamma_{4\psi}$	0.0
	$k_{5\phi}$	0.74730	$\delta_{5\phi}$	0.0	$k_{5\psi}$	0.24088	$\gamma_{5\psi}$	0.0

Table S3: In Amber ff24EXP-GA, the amino acid residues are characterized as glycine-like or alanine-like and assigned the corresponding set of dihedral parameters shown in Table S2. The full set of spectroscopic data and the corresponding Gaussian Ramachandran distributions are available for 14 residues, which are displayed in bold font.

Amino Acid	Letter Code	Dihedral Type
**Gly**	**G**	**G**
**Ala**	**A**	**A**
**Leu**	**L**	**A**
**Val**	**V**	**G**
**Ile**	**I**	**G**
**Phe**	**F**	**G**
His	H	G
Trp	W	G
**Tyr**	**Y**	**G**
**Cys**	**C**	**G**
Met	M	A
**Asn**	**N**	**G**
Gln	Q	A
**Ser**	**S**	**G**
**Thr**	**T**	**G**
**Asp**	**D**	**G**
**Glu**	**E**	**A**
Lys	K	A
**Arg**	**R**	**A**

Table S4: Mesostate populations of guest residues x= G,A,L,V,I,F,Y in GxG peptides as predicted by the Gaussian model, CHARMM36m, Amber ff14SB, and Amber ff24EXP-GA. The values in the parentheses correspond to free energy estimates (in units of k_BT).

GXG	Mesostate	Gaussian	CHARMM36m	Amber ff14SB	Amber ff24EXP-GA
GGG	pPII	0.46 (-2.97)	0.48 (-2.68)	0.40 (-2.50)	0.40 (-2.31)
	βt	0.13 (-2.22)	0.02 (-1.08)	0.06 (-1.45)	0.12 (-2.28)
	$a\beta$	0.01 (-1.07)	0.01 (-0.52)	0.09 (-1.68)	0.03 (-1.28)
	α	0.06 (-1.52)	0.04 (-1.44)	0.05 (-1.39)	0.00 (-0.29)
GAG	pPII	0.59 (-3.62)	0.55 (-3.31)	0.50 (-3.29)	0.60 (-3.74)
	βt	0.16 (-3.18)	0.09 (-2.60)	0.06 (-2.25)	0.14 (-3.03)
	$a\beta$	0.02 (-1.26)	0.12 (-2.63)	0.12 (-2.66)	0.04 (-1.61)
	α	0.02 (-0.87)	0.06 (-2.02)	0.10 (-2.31)	0.03 (-1.28)
GLG	pPII	0.43 (-3.56)	0.51 (-3.33)	0.63 (-3.57)	0.48 (-3.53)
	βt	0.18 (-3.31)	0.13 (-2.88)	0.11 (-2.79)	0.21 (-3.55)
	$a\beta$	0.00 (-1.12)	0.05 (-2.05)	0.05 (-1.96)	0.06 (-2.1)
	α	0.00 (-0.95)	0.07 (-2.17)	0.08 (-2.09)	0.04 (-1.7)
GVG	pPII	0.30 (-3.2)	0.55 (-3.33)	0.52 (-3.33)	0.40 (-2.69)
	βt	0.40 (-3.7)	0.11 (-2.64)	0.09 (-2.49)	0.22 (-3.11)
	$a\beta$	0.02 (-1.94)	0.05 (-2.13)	0.12 (-2.47)	0.09 (-2.39)
	α	0.02 (-1.12)	0.06 (-2.18)	0.1 (-2.19)	0.02 (-1.33)
GIG	pPII	0.28 (-2.9)	0.53 (-3.45)	0.66 (-3.54)	0.30 (-2.78)
	βt	0.10 (-2.48)	0.13 (-2.94)	0.09 (-2.46)	0.11 (-2.54)
	$a\beta$	0.01 (-1.7)	0.09 (-2.59)	0.07 (-2.04)	0.04 (-2.04)
	α	0.06 (-1.82)	0.00 (0.00)	0.07 (-1.98)	0.05 (-1.64)
GFG	pPII	0.35 (-3.14)	0.48 (-3.22)	0.58 (-3.4)	0.42 (-2.67)
	βt	0.34 (-3.94)	0.12 (-2.81)	0.09 (-2.58)	0.22 (-3.15)
	$a\beta$	0.08 (-2.12)	0.10 (-2.44)	0.14 (-2.65)	0.09 (-2.5)
	α	0.05 (-1.89)	0.06 (-1.96)	0.06 (-1.9)	0.01 (-1.06)
GYG	pPII	0.36 (-2.94)	0.46 (-3.14)	0.62 (-3.44)	0.39 (-2.65)
	βt	0.29 (-3.82)	0.11 (-2.65)	0.08 (-2.48)	0.20 (-3.15)
	$a\beta$	0.02 (-1.71)	0.11 (-2.49)	0.17 (-2.83)	0.09 (-2.44)
	α	0.02 (-1.46)	0.08 (-2.21)	0.03 (-1.33)	0.00 (0.00)

Table S5: Mesostate populations of guest residues x= C,N,S,T,D^p,E^p,R in GxG peptides as predicted by the Gaussian model, CHARMM36m, Amber ff14SB, and Amber ff24EXP-GA. The values in the parentheses correspond to free energy estimates (in units of k_BT).

GXG	Mesostate	Gaussian	CHARMM36m	Amber ff14SB	Amber ff24EXP-GA
GCG	pPII	0.20 (-2.65)	0.52 (-3.24)	0.59 (-3.47)	0.47 (-2.75)
	βt	0.17 (-3.14)	0.10 (-2.67)	0.07 (-2.36)	0.14 (-2.97)
	$a\beta$	0.02 (-1.48)	0.08 (-2.34)	0.11 (-2.46)	0.06 (-2.30)
	α	0.01 (-1.32)	0.05 (-1.99)	0.08 (-1.97)	0.01 (-1.17)
GNG	pPII	0.33 (-3.25)	0.45 (-3.18)	0.54 (-3.42)	0.39 (-2.65)
	βt	0.23 (-3.26)	0.13 (-2.88)	0.09 (-2.60)	0.19 (-3.16)
	$a\beta$	0.05 (-2.08)	0.06 (-2.10)	0.1 (-2.33)	0.06 (-2.33)
	α	0.01 (-0.85)	0.05 (-2.04)	0.06 (-1.88)	0.01 (-1.20)
GSG	pPII	0.33 (-3.04)	0.37 (-2.99)	0.44 (-3.17)	0.36 (-2.64)
	βt	0.30 (-3.65)	0.09 (-2.62)	0.06 (-2.22)	0.13 (-2.92)
	$a\beta$	0.01 (-1.61)	0.19 (-2.87)	0.22 (-3.09)	0.09 (-2.44)
	α	0.01 (-1.29)	0.05 (-2.01)	0.08 (-2.00)	0.11 (-1.17)
GTG	pPII	0.16 (-2.50)	0.46 (-3.19)	0.45 (-3.24)	0.34 (-2.62)
	βt	0.32 (-3.58)	0.11 (-2.71)	0.08 (-2.41)	0.16 (-3.02)
	$a\beta$	0.03 (-1.72)	0.21 (-3.00)	0.22 (-3.03)	0.08 (-2.36)
	α	0.01 (-1.44)	0.03 (-1.85)	0.07 (-1.89)	0.02 (-1.40)
GD^pG	pPII	0.08 (-2.22)	0.36 (-2.99)	0.48 (-3.36)	0.29 (-2.60)
	βt	0.16 (-2.86)	0.17 (-3.19)	0.09 (-2.57)	0.15 (-3.00)
	$a\beta$	0.08 (-2.23)	0.12 (-2.75)	0.12 (-2.50)	0.09 (-2.48)
	α	0.01 (-1.25)	0.03 (-1.86)	0.05 (-1.69)	0.01 (-1.08)
GE^pG	pPII	0.39 (-3.23)	0.36 (-3.05)	0.48 (-3.27)	0.41 (-3.33)
	βt	0.25 (-3.86)	0.08 (-2.59)	0.08 (-2.51)	0.17 (-3.3)
	$a\beta$	0.05 (-1.85)	0.17 (-2.98)	0.16 (-2.74)	0.17 (-2.8)
	α	0.02 (-1.87)	0.10 (-2.7)	0.07 (-1.92)	0.03 (-1.34)
GRG	pPII	0.42 (-3.32)	0.46 (-3.17)	0.51 (-3.34)	0.43 (-3.38)
	βt	0.21 (-3.52)	0.13 (-2.9)	0.08 (-2.55)	0.17 (-3.31)
	$a\beta$	0.02 (-1.5)	0.11 (-2.62)	0.15 (-2.65)	0.13 (-2.64)
	α	0.00 (-2.28)	0.05 (-1.95)	0.10 (-2.21)	0.05(-1.9)

Table S6: Comparison of experimental and MD-derived J-coupling constant values for A2, A3, and A4 in Ala_5 alongside experimental (s_{J_i}) and statistical (s_i) uncertainties defined in *Methods*. Statistical uncertainties associated with the three Karplus parameters, see Eq.(11), are taken from Hu and Bax.[S1]

Residue	J-coupling constants (Hz)							
	Type	Exp.	C36m		ff14SB		ff24EXP-GA	
		J $\pm s_{J_i}$	J	s_i	J	s_i	J	s_i
A2	$^3J(H^N, H^{C_\alpha})$	5.59±0.03	6.139	0.129	5.856	0.124	5.421	0.126
	$^3J(H^N, C')$	1.13±0.00	1.251	0.111	1.173	0.121	1.351	0.113
	$^3J(H^{C_\alpha}, C')$	1.85±0.05	1.848	0.105	1.682	0.100	1.624	0.103
	$^3J(H^N, C_\beta)$	2.30±0.00	1.765	0.034	1.941	0.034	2.007	0.035
	$^1J(N, C_\alpha)$	11.36±0.03	11.345	0.030	11.543	0.030	11.504	0.030
A3	$^3J(H^N, H^{C_\alpha})$	5.74±0.02	6.153	0.127	5.985	0.120	5.513	0.126
	$^3J(H^{C_\alpha}, C')$	1.86±0.05	1.903	0.104	1.954	0.099	1.665	0.105
	$^3J(H^N, C_\beta)$	2.24±0.01	1.804	0.036	1.926	0.037	2.000	0.037
	$^1J(N, C_\alpha)$	11.26±0.03	11.181	0.030	11.297	0.030	11.320	0.030
A4	$^3J(H^N, H^{C_\alpha})$	5.98±0.02	6.374	0.125	6.213	0.122	5.784	0.126
	$^3J(H^N, C')$	1.15±0.02	1.223	0.111	1.103	0.119	1.264	0.105
	$^3J(H^{C_\alpha}, C')$	1.89±0.02	2.036	0.092	1.686	0.090	1.820	0.093
	$^3J(H^N, C_\beta)$	2.14±0.00	1.687	0.036	1.830	0.035	1.913	0.038
	$^1J(N, C_\alpha)$	11.25±0.02	11.235	0.020	11.386	0.020	11.257	0.020

Supporting Figures

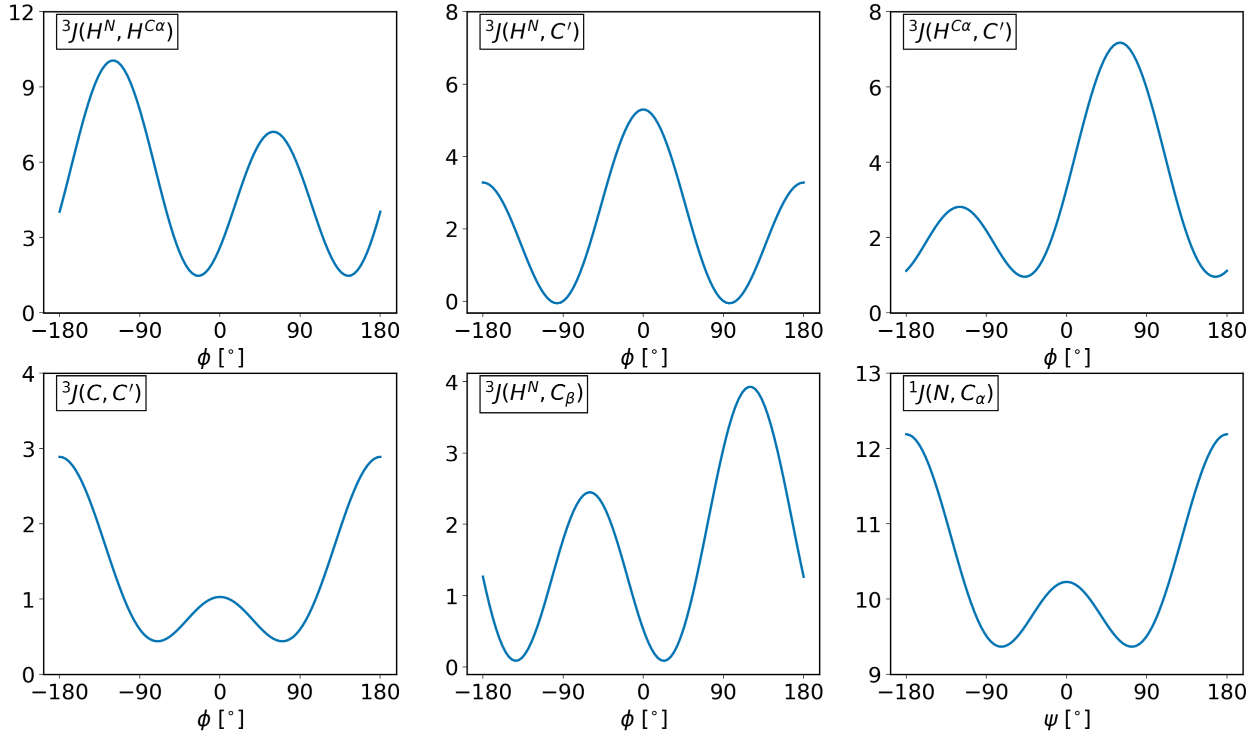

Figure S1: Plots of J-coupling constants used in our analysis. $^3J(C,C')$ was used only for GGG and $^3J(H^N,C_\beta)$ was used for all GxG peptides except GGG[S1–S3] (the units are in Hz).

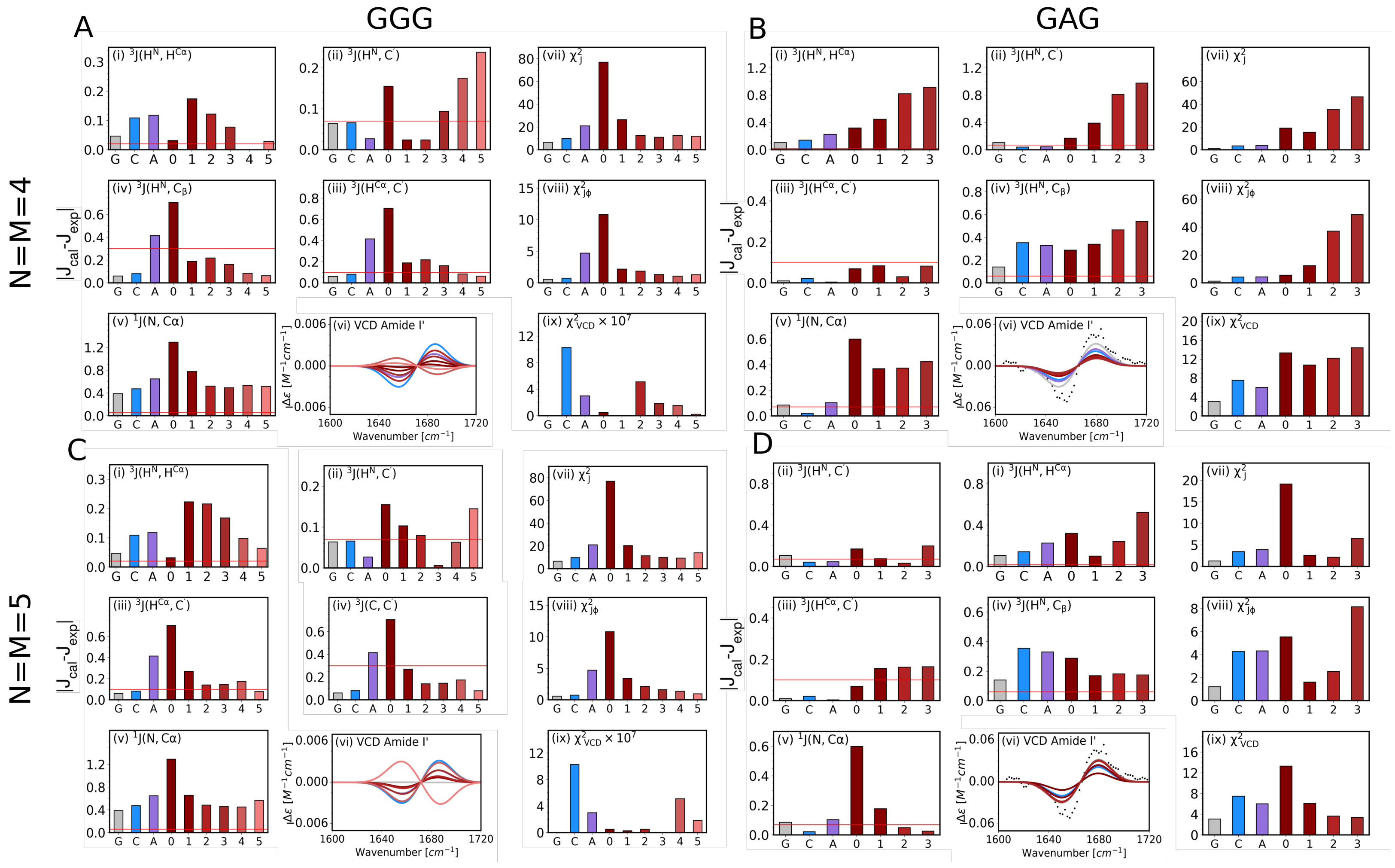

Figure S2: Comparison of J-coupling constants and the amide I' profiles during the IBI performed by using (A, B) $N = 4$ and (C, D) $M = 5$ terms in ϕ- and ψ-dependent dihedral potential for guest residues in (A, C) GGG and (B, D) GAG. The Gaussian model, CHARMM36m and Amber ff14SB force fields are labeled as G, C, and A, respectively. The dotted lines in the amide I' profiles for guest alanine in GAG (panels B-vi and D-vi) represent the experimental data. Because GGG is achiral, the corresponding experimental amide I' profile vanishes. The x-axis labels 0, 1, 2, 3 correspond to IBI iteration numbers.

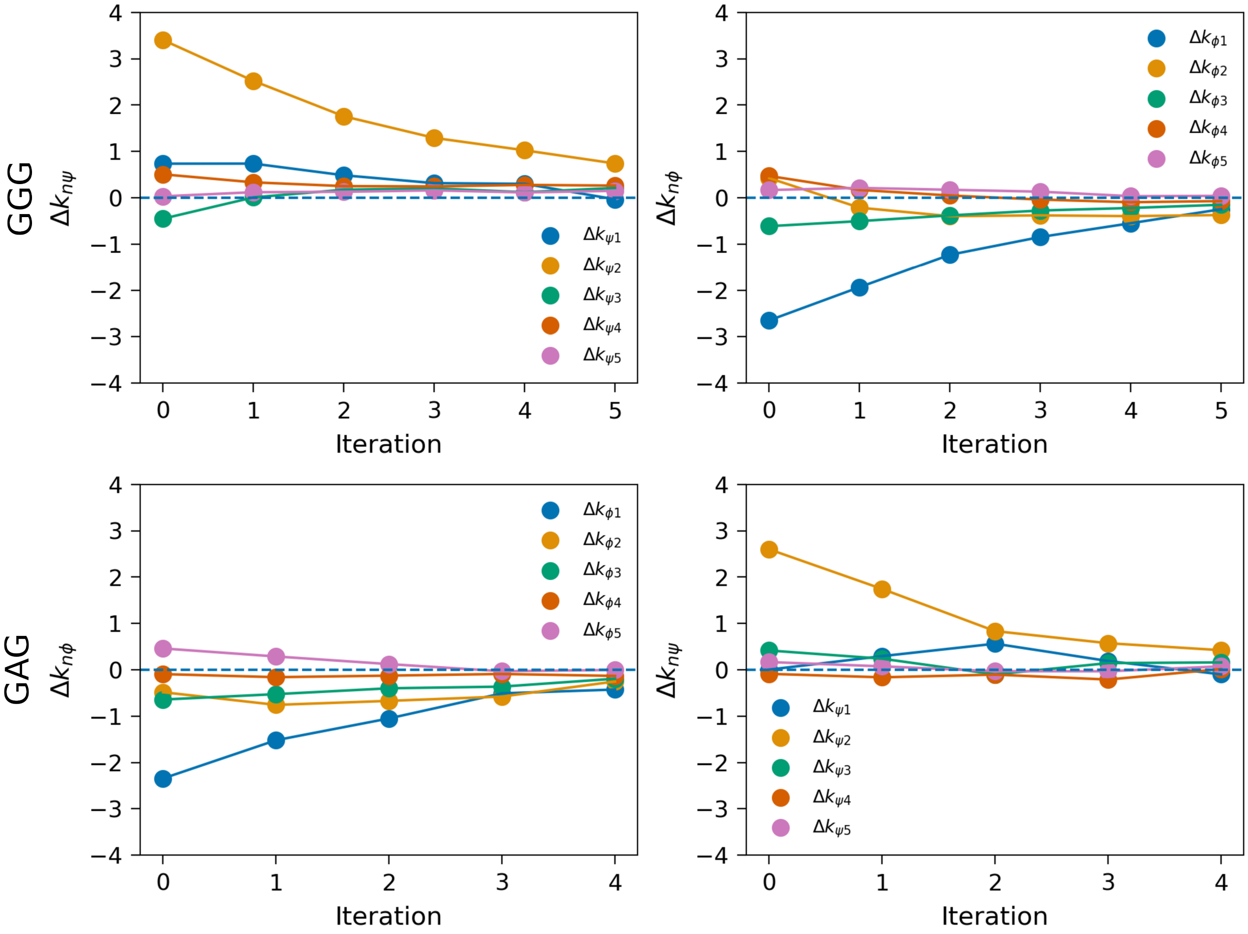

Figure S3: Convergence of the $\Delta k_{n\phi}$ and $\Delta k_{m\psi}$ parameters during the IBI procedure using $N = 5$ terms in the cosine expansions for guest residues in (A, B) GGG and (C, D) GAG.

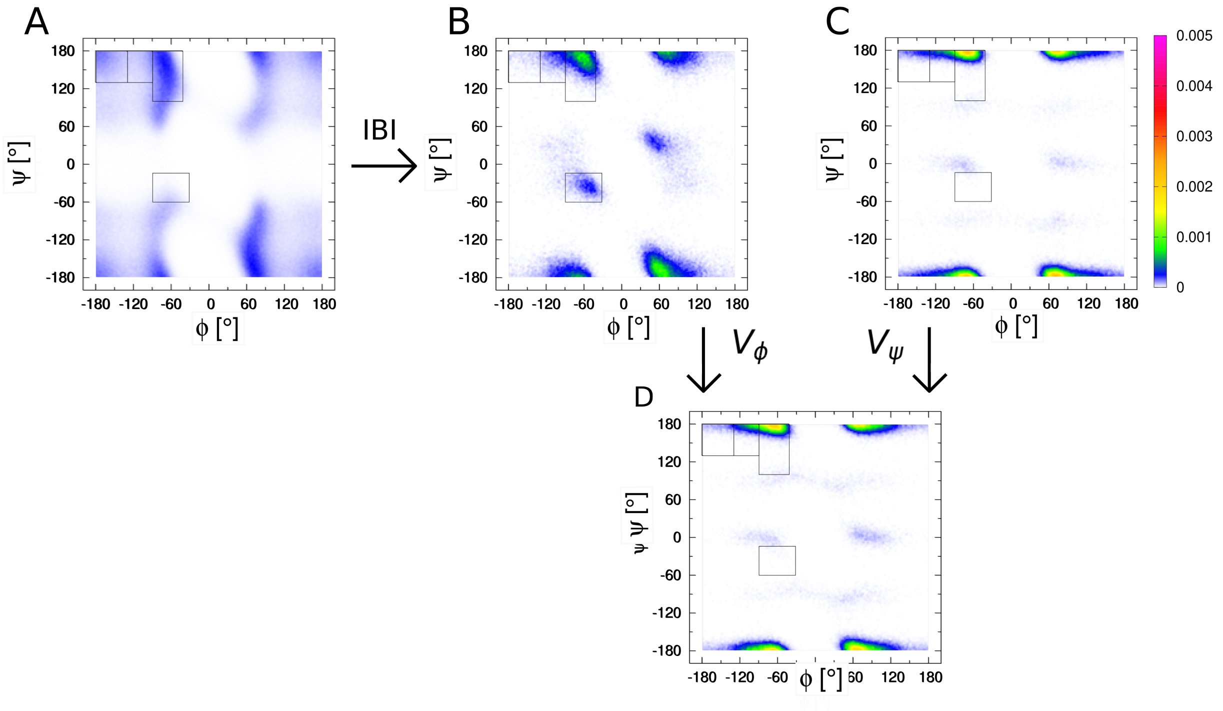

Figure S4: Optimization procedure for the guest glycine residue in GGG. (A) Initial distribution, (B) IBI-optimized distribution after 5 iterations, (C) manually optimized distribution, and (D) final Ramachandran distribution that combines the potential for ϕ from (B) with the potential for ψ from (C).

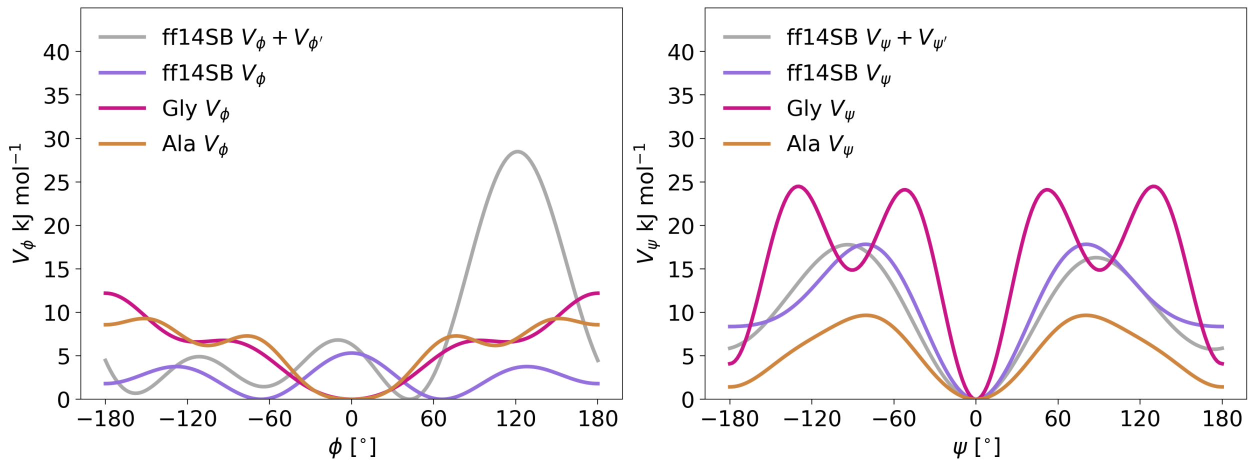

Figure S5: Backbone dihedral potentials corresponding to Amber ff 14SB, and the optimized backbone potentials for glycine and alanine. The new backbone dihedral parameter sets, when combined with all the Amber ff14SB force field parameters except the backbone dihedral, will are referred to as Amber ff24EXP-G and Amber ff24EXP-A respectively based on the backbone parameters derived using glycine and alanine respectively. The prime potentials ($V_{\phi'}$ and $V_{\phi'}$) were set to zero in the modified force fields.

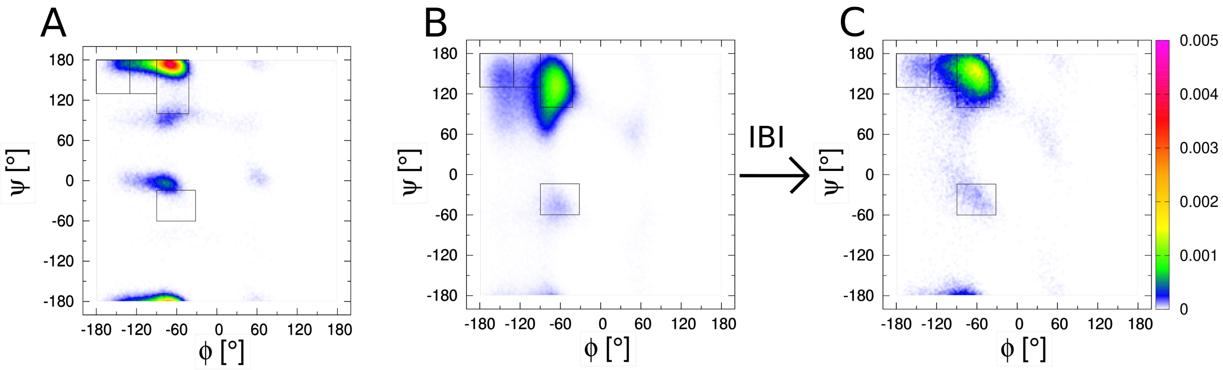

Figure S6: (A) GAG Ramachandran distribution of GAG using dihedral parameters with glycine. Since this parameter set performed poorly for alanine, we modified the backbone dihedral potential using IBI method, with alanine's Gaussian Ramachandran distribution as reference. (B) Initial distribution and (C) final IBI-optimized Ramachandran distribution after 2 iterations.

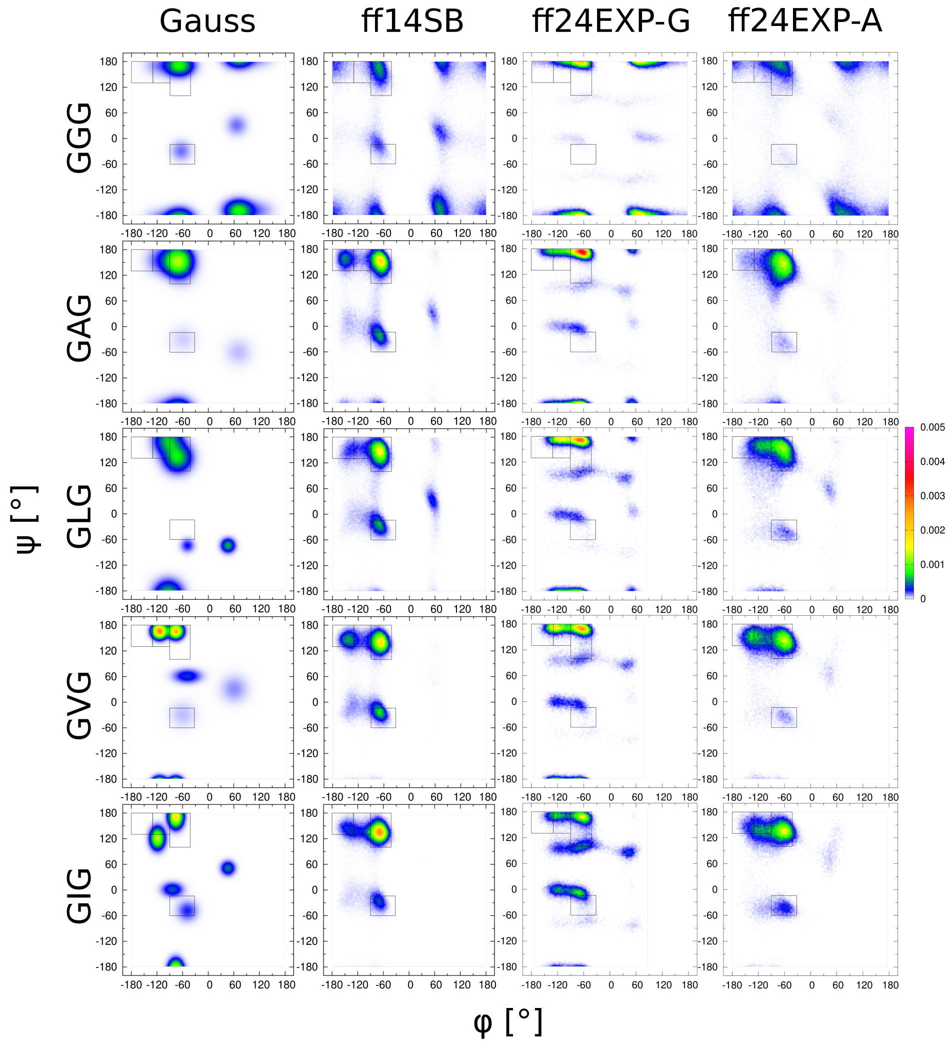

Figure S7: Ramachandran distributions of aliphatic guest amino acid residues in GxG (x=G,A,L,V,I) derived within Amber ff14SB, glycine-optimized Amber ff24EXP-G and alanine-optimized Amber ff24EXP-A.

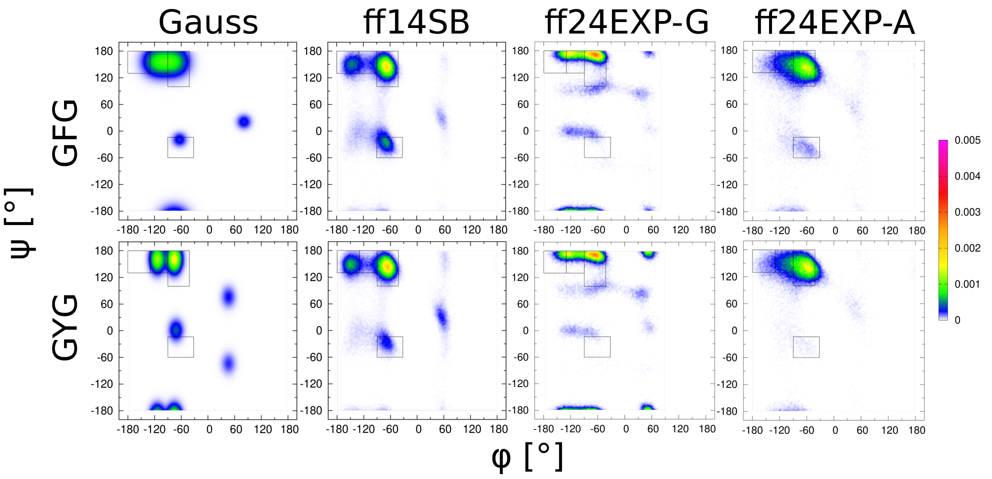

Figure S8: Ramachandran distributions of aromatic guest amino acid residues in GxG (x=F,Y) derived within Amber ff14SB, glycine-optimized Amber ff24EXP-G and alanine-optimized Amber ff24EXP-A.

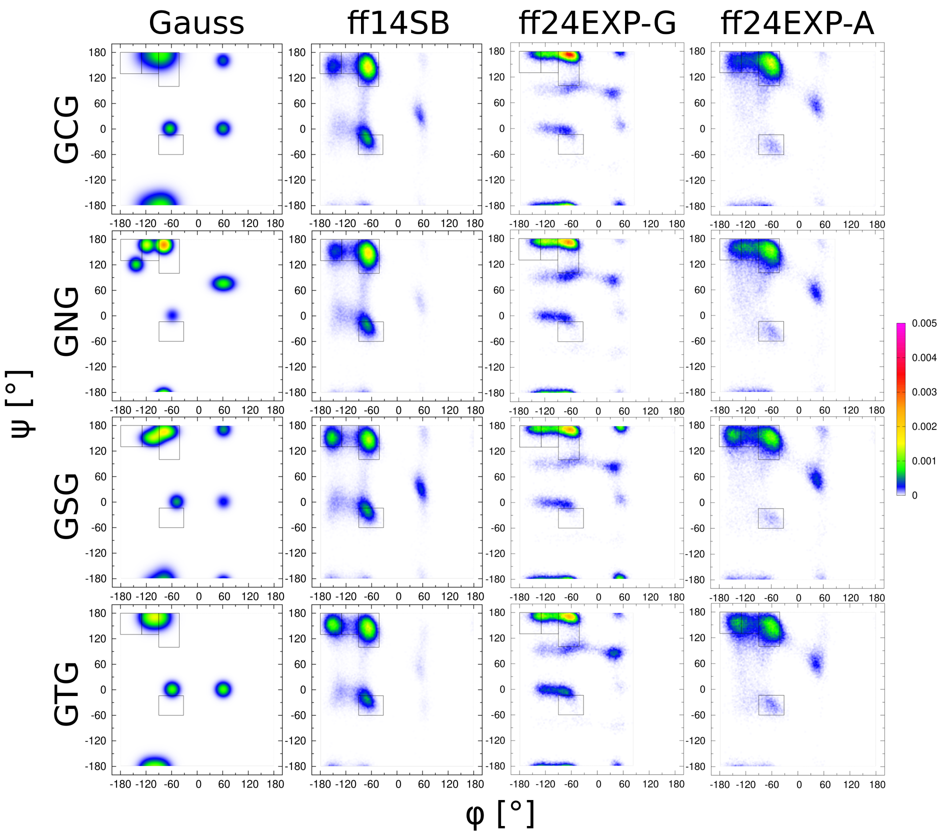

Figure S9: Ramachandran distributions of polar guest amino acid residues in GxG (x=C,N,S,T) derived within Amber ff14SB, glycine-optimized Amber ff24EXP-G and alanine-optimized Amber ff24EXP-A.

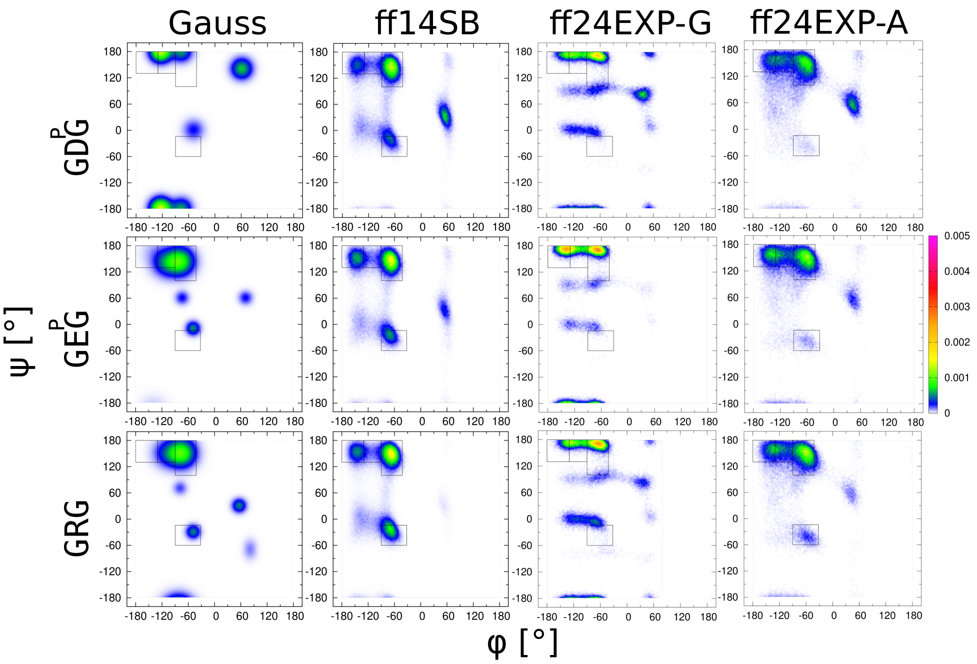

Figure S10: Ramachandran distributions of charged guest amino acid residues in GxG (x=D^P,E^P and R) derived within Amber ff14SB, glycine-optimized Amber ff24EXP-G and alanine-optimized Amber ff24EXP-A.

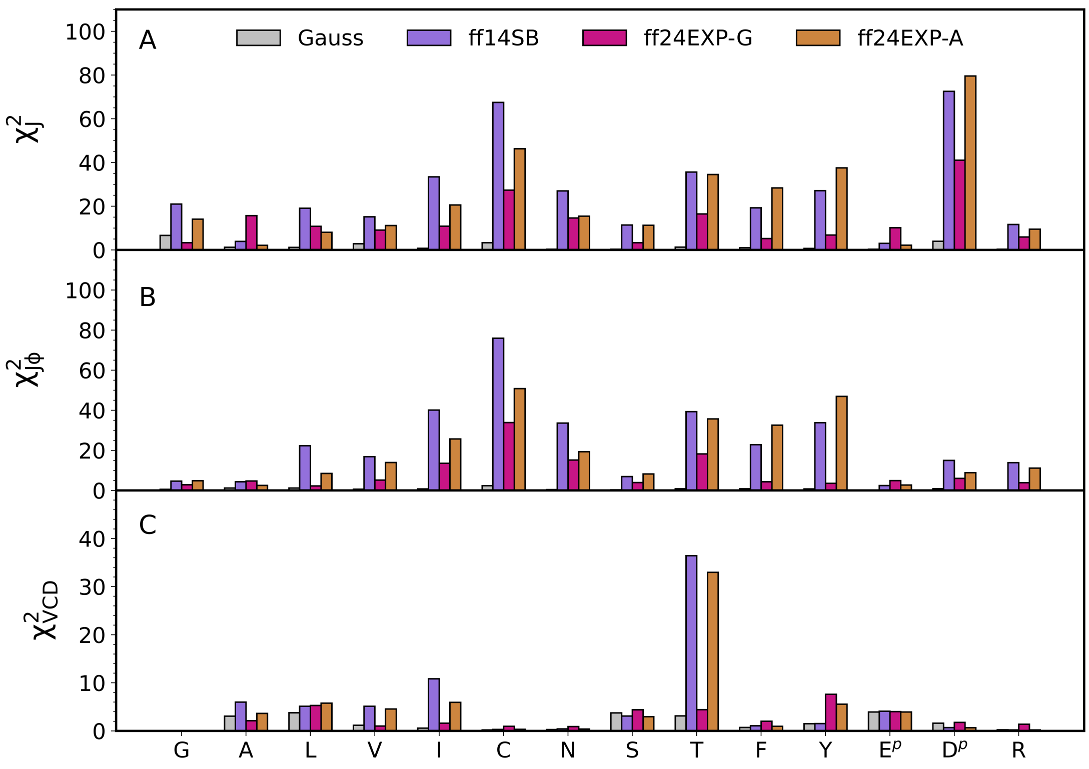

Figure S11: Summary of χ^2_J, $\chi^2_{J\phi}$, and χ^2_{VCD} for guest residue x=G, A, L, V, I, F, Y, C, N, S, T, D^P, E^P and R in GxG peptides for the Gaussian model, Amber ff14SB, Amber ff24EXP-G and Amber ff24EXP-A.

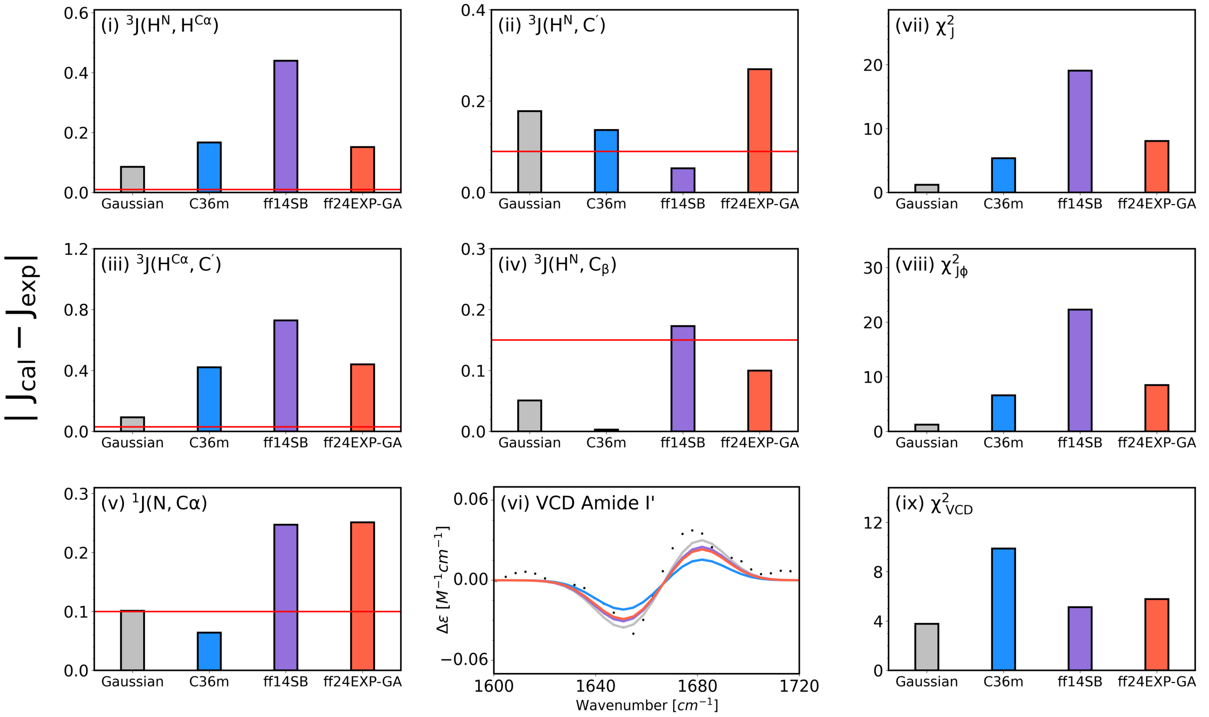

Figure S12: Comparison of experimental and computed J-coupling constants and VCD amide I' profiles of guest L in GLG for the Gaussian model, CHARMM36m, Amber ff14SB and Amber ff24EXP-GA. (i-v) Absolute differences between calculated and experimental values of the five J-coupling constants for the Gaussian model and the three MD force fields. Red lines correspond to experimental uncertainties. (vi) VCD amide I' profiles computed using the Gaussian model and the three MD force fields in comparison to experimental data. (vii-ix) Reduced ${\chi^2}_J$, ${\chi^2}_{J\phi}$ and χ^2_{VCD} values for the Gaussian model and the three MD force fields.

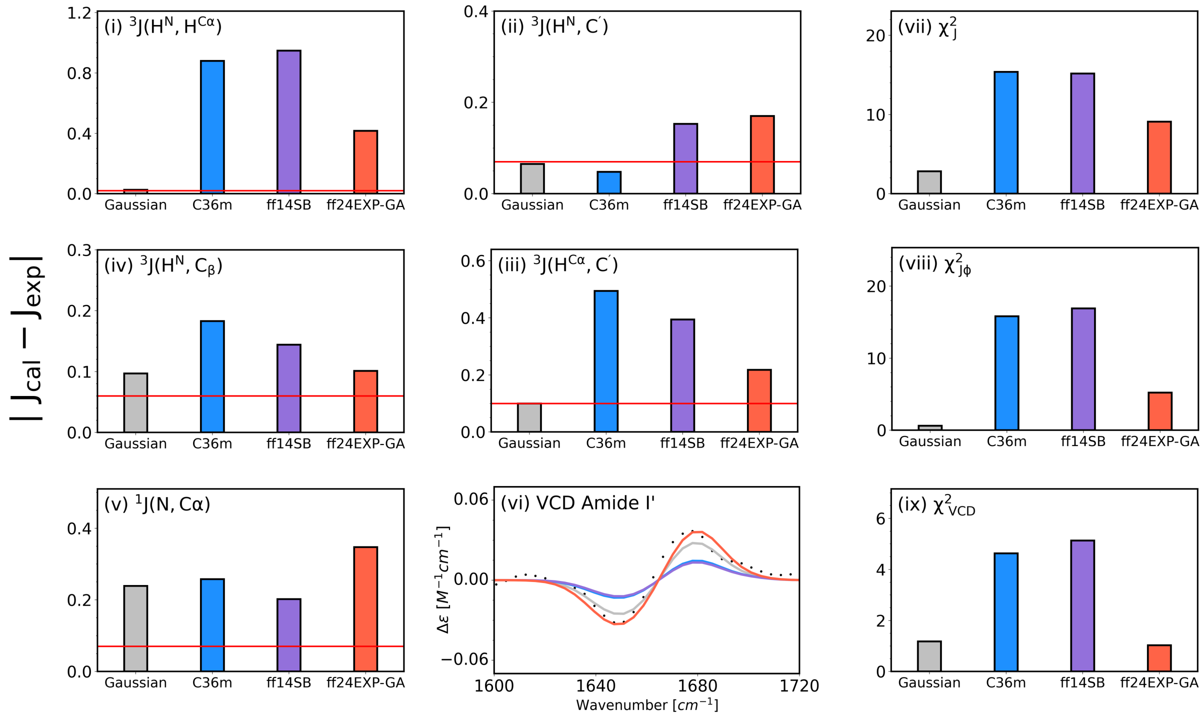

Figure S13: Comparison of experimental and computed J-coupling constants and VCD amide I' profiles of guest V in GVG for the Gaussian model, CHARMM36m, Amber ff14SB and Amber ff24EXP-GA. (i-v) Absolute differences between calculated and experimental values of the five J-coupling constants for the Gaussian model and the three MD force fields. Red lines correspond to experimental uncertainties. (vi) VCD amide I' profiles computed using the Gaussian model and the three MD force fields in comparison to experimental data. (vii-ix) Reduced ${\chi^2}_J$, ${\chi^2}_{J\phi}$ and χ^2_{VCD} values for the Gaussian model and the three MD force fields.

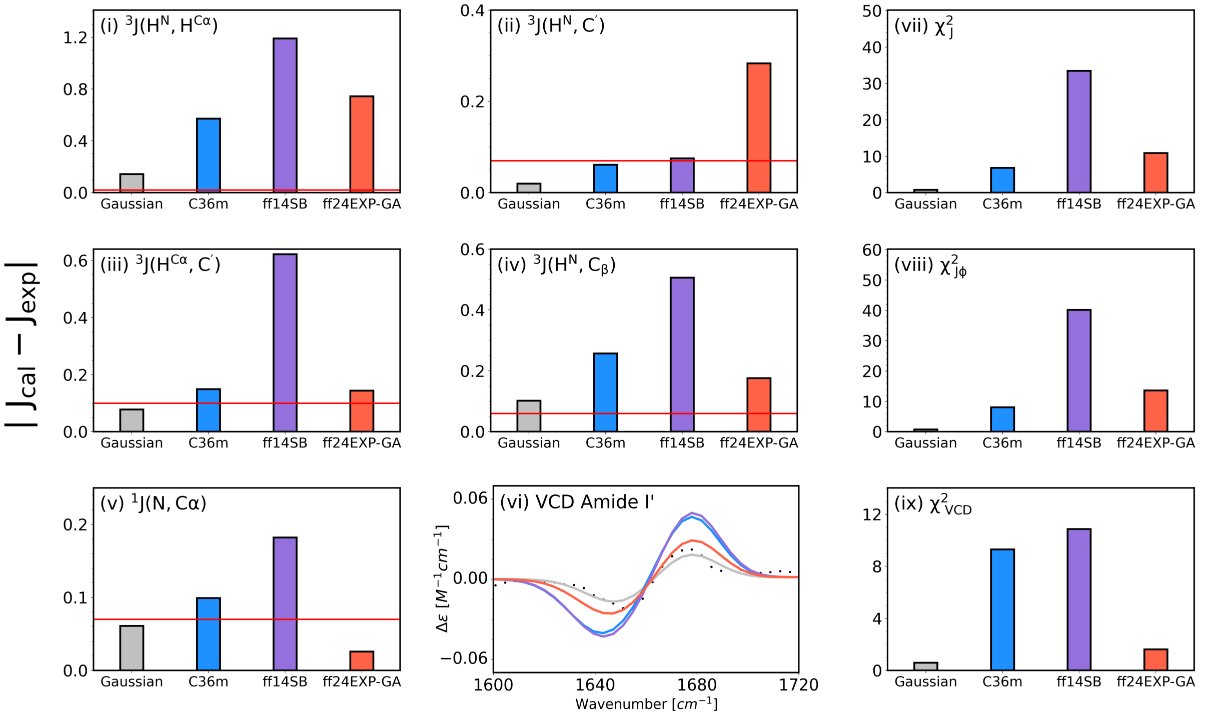

Figure S14: Comparison of experimental and computed J-coupling constants and VCD amide I' profiles of guest I in GIG for the Gaussian model, CHARMM36m, Amber ff14SB and Amber ff24EXP-GA. (i-v) Absolute differences between calculated and experimental values of the five J-coupling constants for the Gaussian model and the three MD force fields. Red lines correspond to experimental uncertainties. (vi) VCD amide I' profiles computed using the Gaussian model and the three MD force fields in comparison to experimental data. (vii-ix) Reduced ${\chi^2}_J$, ${\chi^2}_{J\phi}$ and χ^2_{VCD} values for the Gaussian model and the three MD force fields.

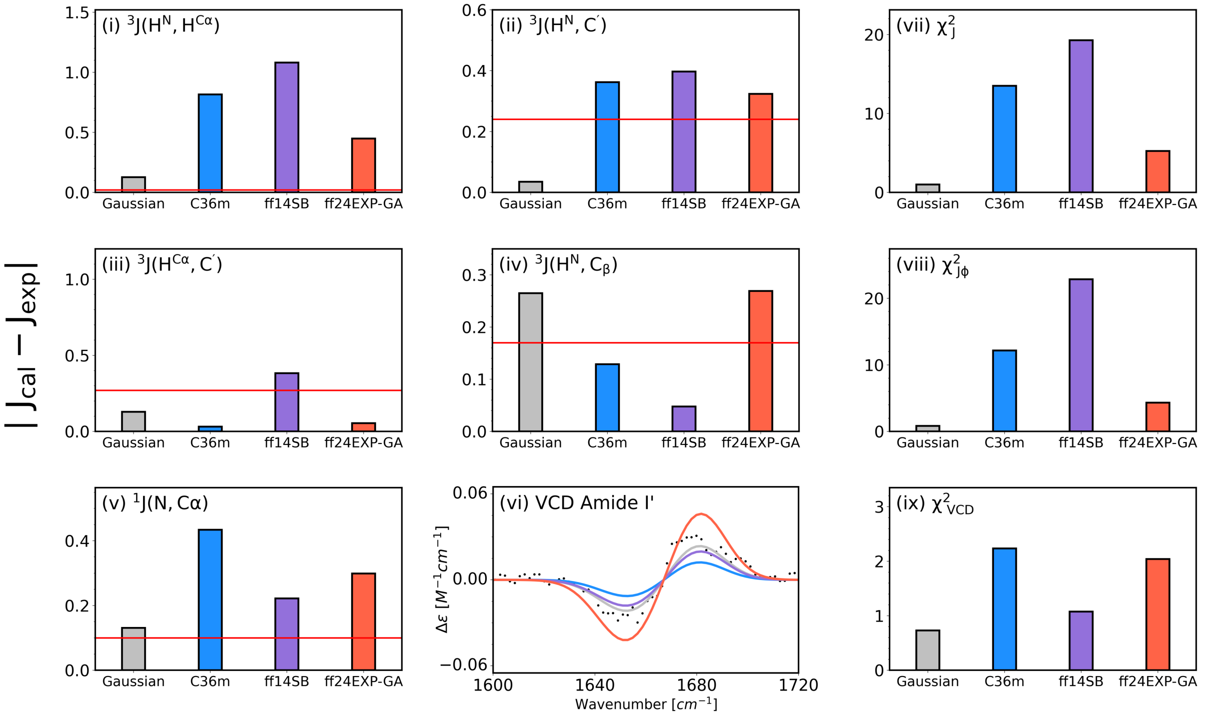

Figure S15: Comparison of experimental and computed J-coupling constants and VCD amide I' profiles of guest F in GFG for the Gaussian model, CHARMM36m, Amber ff14SB and Amber ff24EXP-GA. (i-v) Absolute differences between calculated and experimental values of the five J-coupling constants for the Gaussian model and the three MD force fields. Red lines correspond to experimental uncertainties. (vi) VCD amide I' profiles computed using the Gaussian model and the three MD force fields in comparison to experimental data. (vii-ix) Reduced ${\chi^2}_J$, ${\chi^2}_{J\phi}$ and χ^2_{VCD} values for the Gaussian model and the three MD force fields.

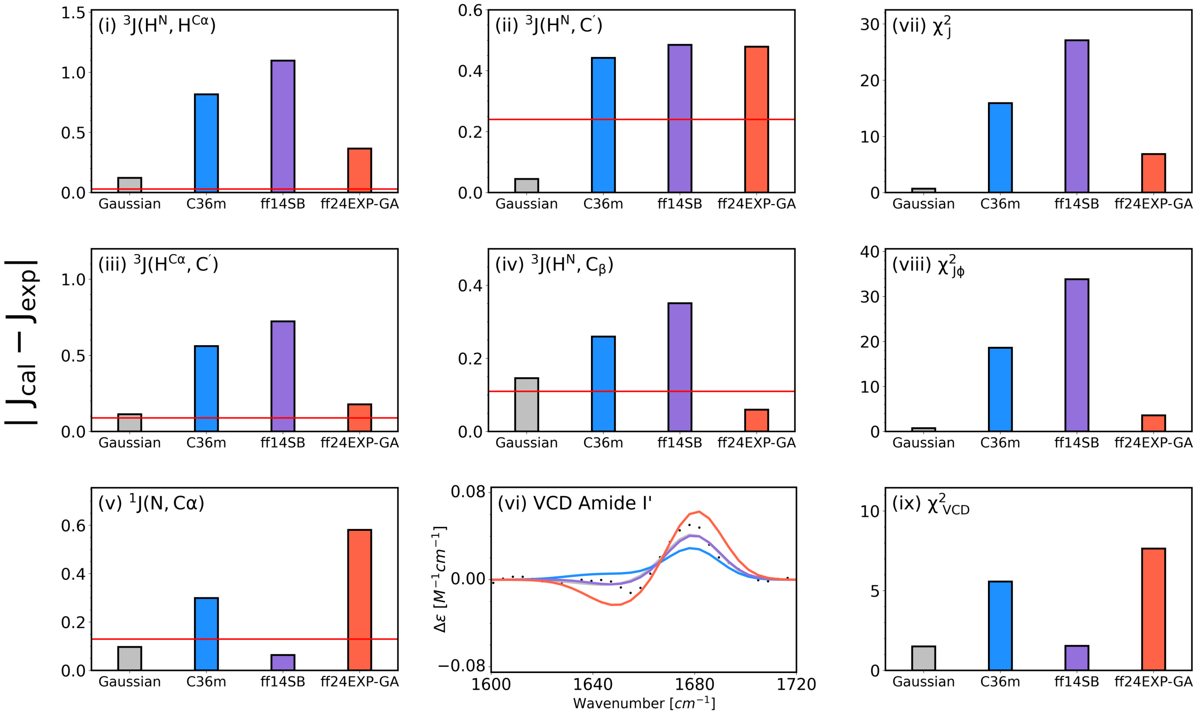

Figure S16: Comparison of experimental and computed J-coupling constants and VCD amide I' profiles of guest Y in GYG for the Gaussian model, CHARMM36m, Amber ff14SB and Amber ff24EXP-GA. (i-v) Absolute differences between calculated and experimental values of the five J-coupling constants for the Gaussian model and the three MD force fields. Red lines correspond to experimental uncertainties. (vi) VCD amide I' profiles computed using the Gaussian model and the three MD force fields in comparison to experimental data. (vii-ix) Reduced ${\chi^2}_J$, ${\chi^2}_{J\phi}$ and χ^2_{VCD} values for the Gaussian model and the three MD force fields.

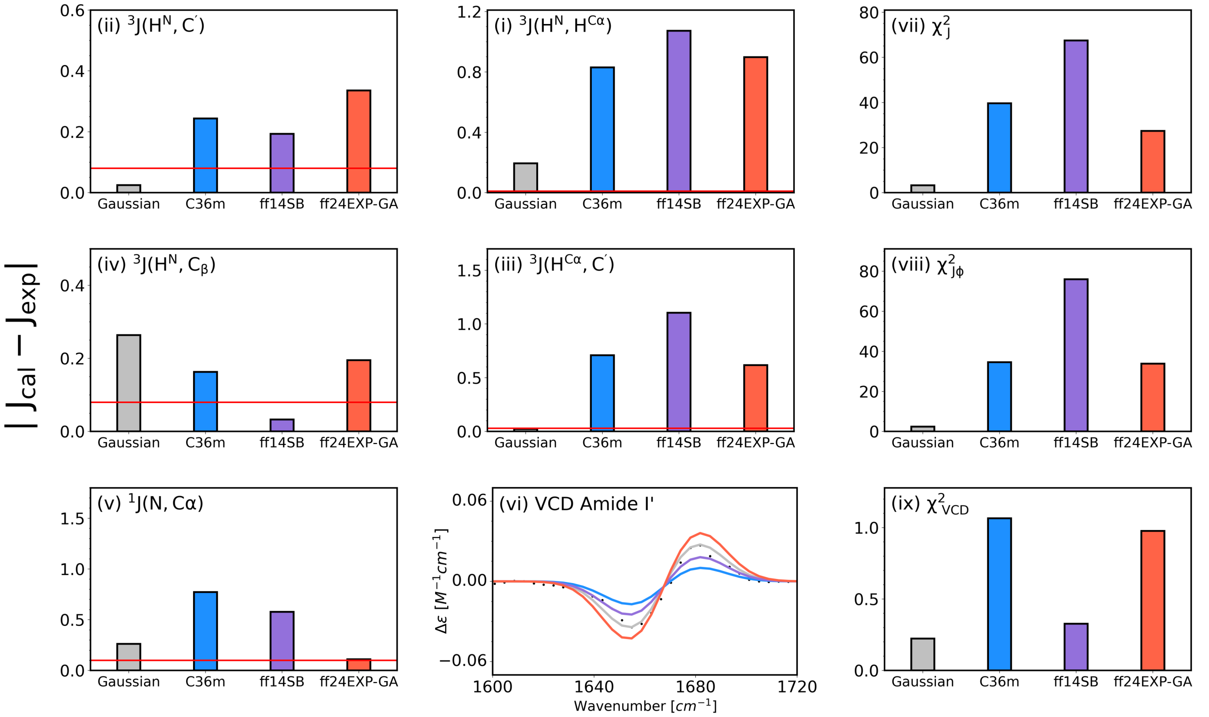

Figure S17: Comparison of experimental and computed J-coupling constants and VCD amide I' profiles of guest C in GCG for the Gaussian model, CHARMM36m, Amber ff14SB and Amber ff24EXP-GA. (i-v) Absolute differences between calculated and experimental values of the five J-coupling constants for the Gaussian model and the three MD force fields. Red lines correspond to experimental uncertainties. (vi) VCD amide I' profiles computed using the Gaussian model and the three MD force fields in comparison to experimental data. (vii-ix) Reduced ${\chi^2}_J$, ${\chi^2}_{J\phi}$ and χ^2_{VCD} values for the Gaussian model and the three MD force fields.

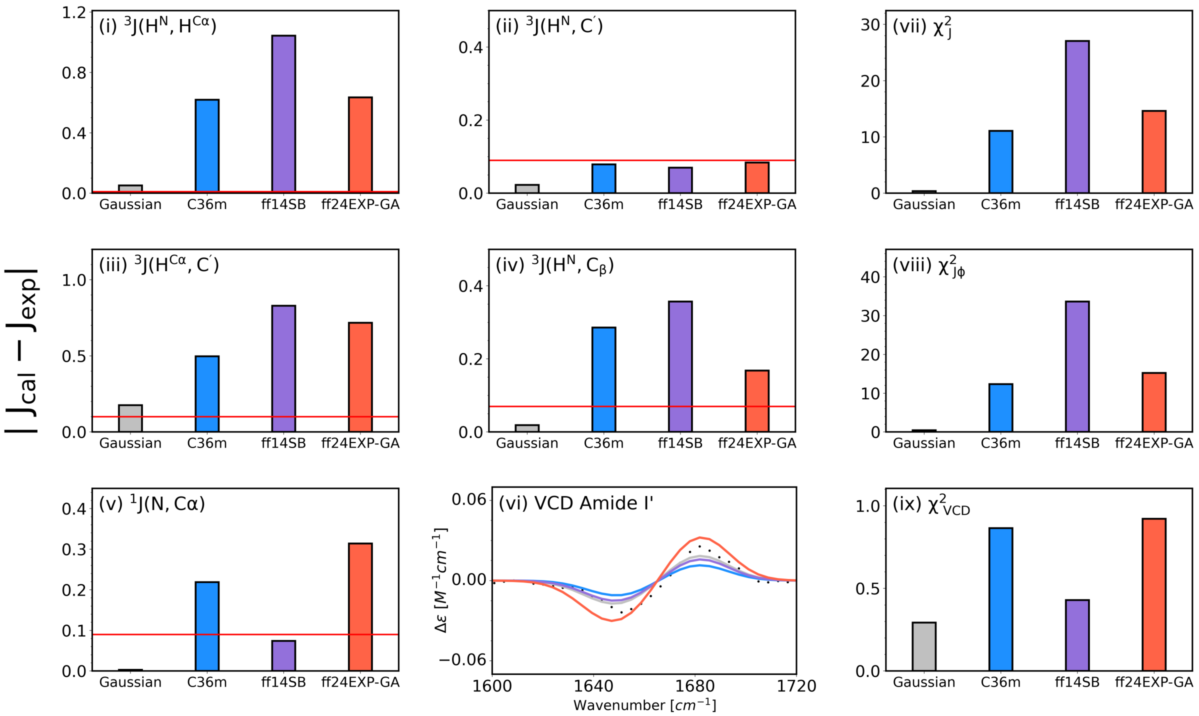

Figure S18: Comparison of experimental and computed J-coupling constants and VCD amide I' profiles of guest N in GNG for the Gaussian model, CHARMM36m, Amber ff14SB and Amber ff24EXP-GA. (i-v) Absolute differences between calculated and experimental values of the five J-coupling constants for the Gaussian model and the three MD force fields. Red lines correspond to experimental uncertainties. (vi) VCD amide I' profiles computed using the Gaussian model and the three MD force fields in comparison to experimental data. (vii-ix) Reduced ${\chi^2}_J$, ${\chi^2}_{J\phi}$ and χ^2_{VCD} values for the Gaussian model and the three MD force fields.

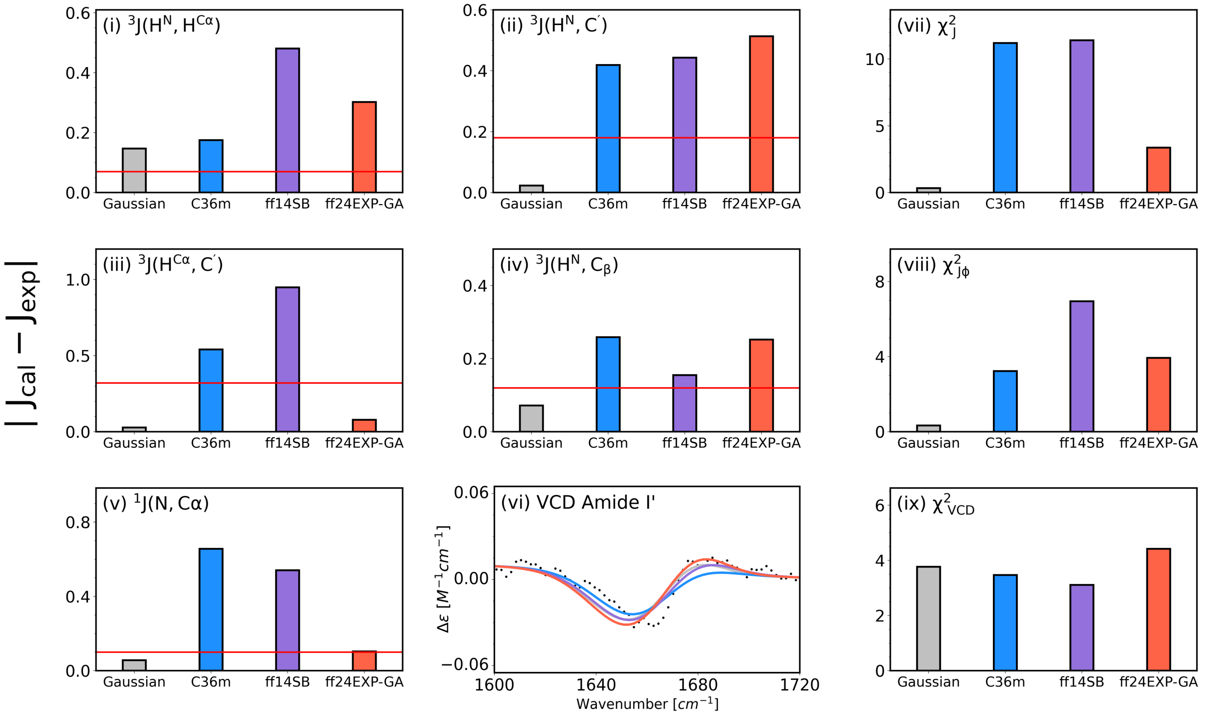

Figure S19: Comparison of experimental and computed J-coupling constants and VCD amide I' profiles of guest S in GSG for the Gaussian model, CHARMM36m, Amber ff14SB and Amber ff24EXP-GA. (i-v) Absolute differences between calculated and experimental values of the five J-coupling constants for the Gaussian model and the three MD force fields. Red lines correspond to experimental uncertainties. (vi) VCD amide I' profiles computed using the Gaussian model and the three MD force fields in comparison to experimental data. (vii-ix) Reduced ${\chi^2}_J$, ${\chi^2}_{J\phi}$ and χ^2_{VCD} values for the Gaussian model and the three MD force fields.

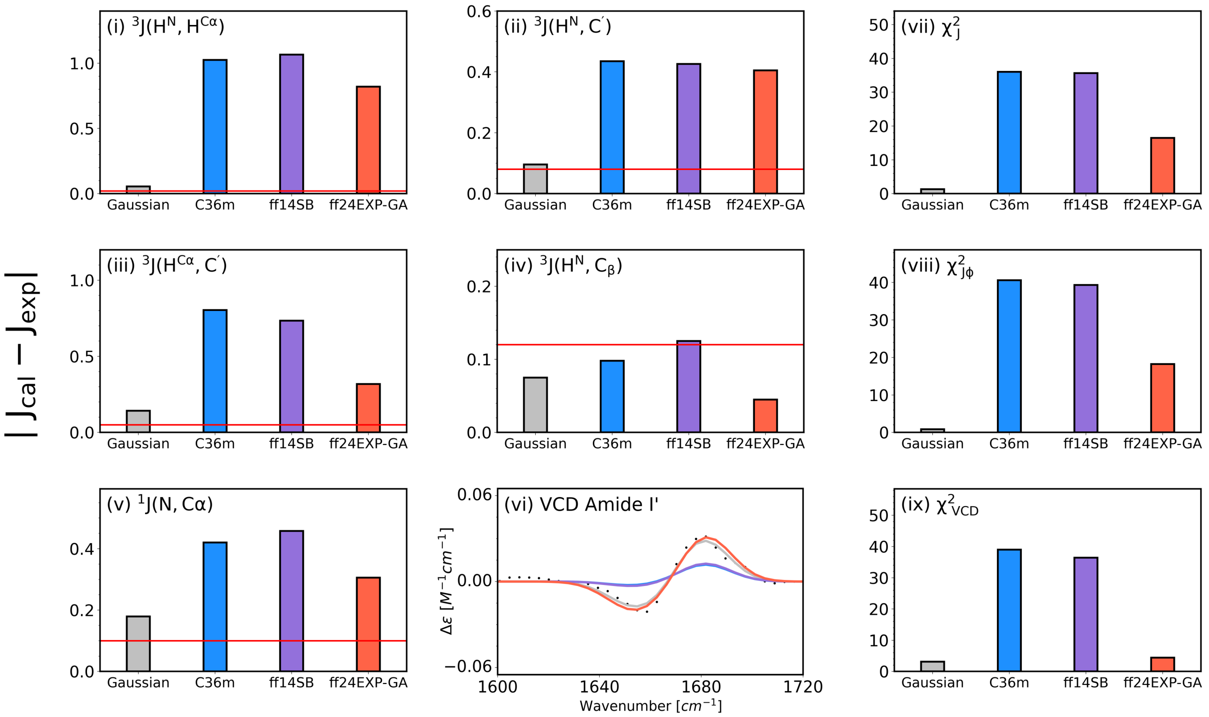

Figure S20: Comparison of experimental and computed J-coupling constants and VCD amide I' profiles of guest T in GTG for the Gaussian model, CHARMM36m, Amber ff14SB and Amber ff24EXP-GA. (i-v) Absolute differences between calculated and experimental values of the five J-coupling constants for the Gaussian model and the three MD force fields. Red lines correspond to experimental uncertainties. (vi) VCD amide I' profiles computed using the Gaussian model and the three MD force fields in comparison to experimental data. (vii-ix) Reduced ${\chi^2}_J$, ${\chi^2}_{J\phi}$ and χ^2_{VCD} values for the Gaussian model and the three MD force fields.

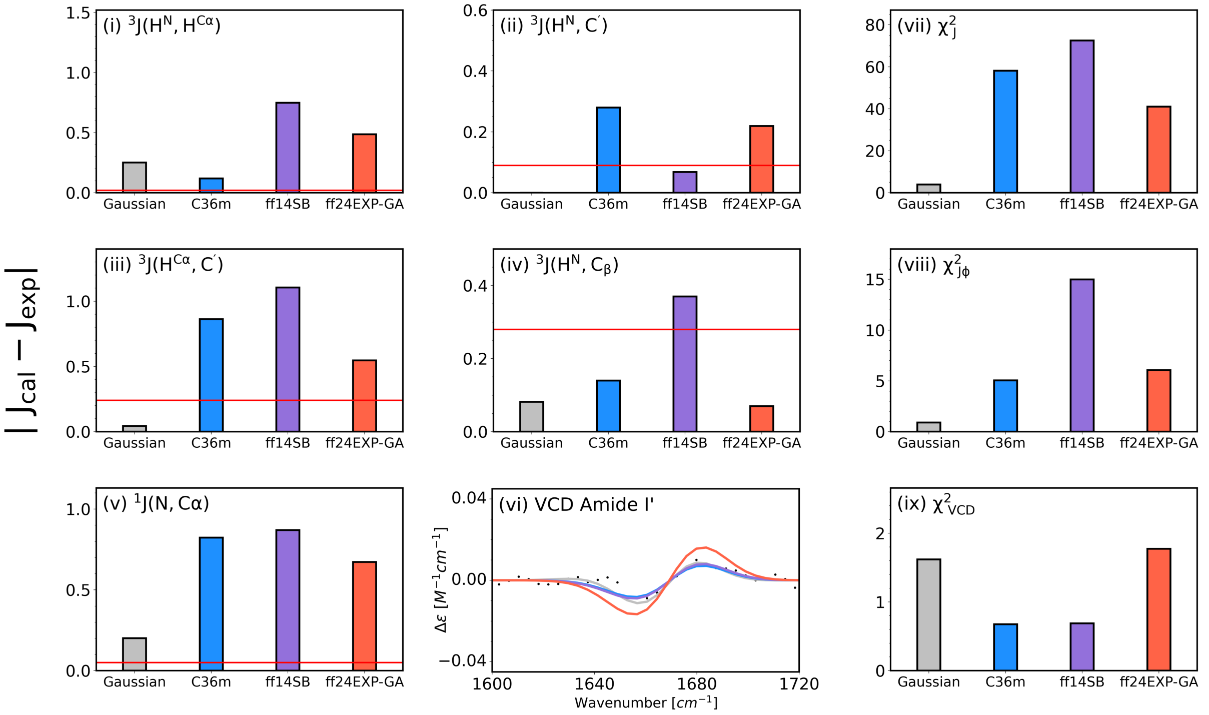

Figure S21: Comparison of experimental and computed J-coupling constants and VCD amide I' profiles of guest D^P in GD^PG for the Gaussian model, CHARMM36m, Amber ff14SB and Amber ff24EXP-GA. (i-v) Absolute differences between calculated and experimental values of the five J-coupling constants for the Gaussian model and the three MD force fields. Red lines correspond to experimental uncertainties. (vi) VCD amide I' profiles computed using the Gaussian model and the three MD force fields in comparison to experimental data. (vii-ix) Reduced ${\chi^2}_J$, ${\chi^2}_{J\phi}$ and χ^2_{VCD} values for the Gaussian model and the three MD force fields.

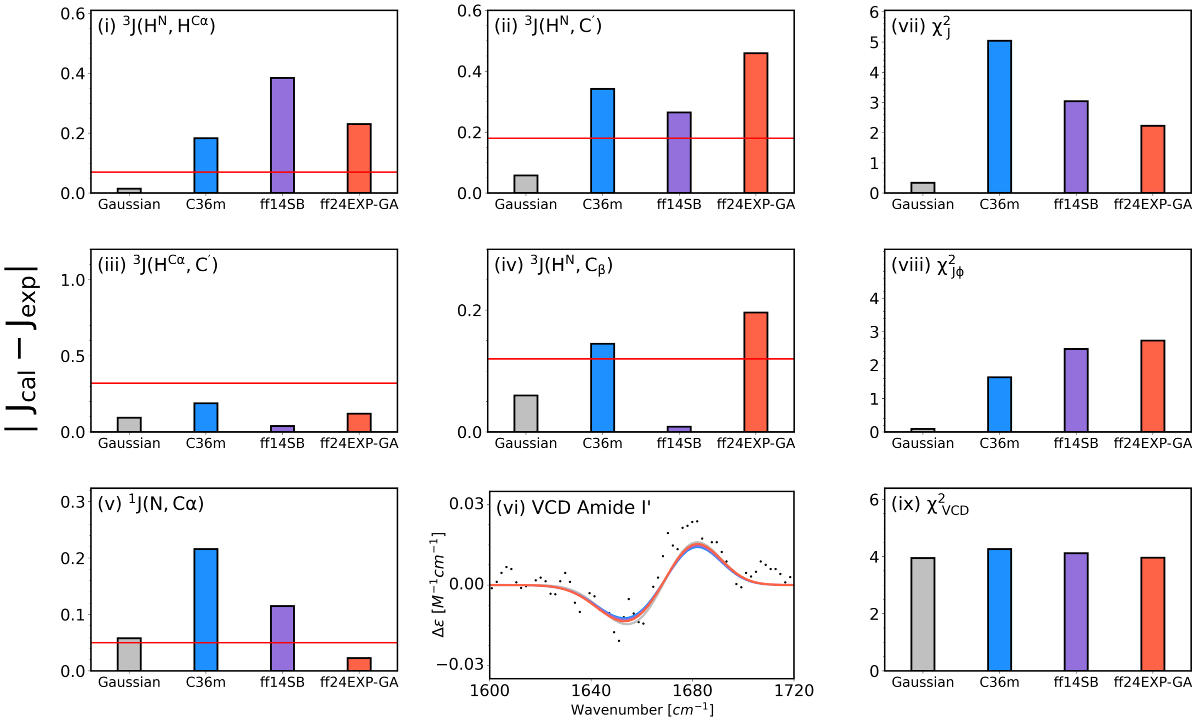

Figure S22: Comparison of experimental and computed J-coupling constants and VCD amide I' profiles of guest E^P in GE^PG for the Gaussian model, CHARMM36m, Amber ff14SB and Amber ff24EXP-GA. (i-v) Absolute differences between calculated and experimental values of the five J-coupling constants for the Gaussian model and the three MD force fields. Red lines correspond to experimental uncertainties. (vi) VCD amide I' profiles computed using the Gaussian model and the three MD force fields in comparison to experimental data. (vii-ix) Reduced ${\chi^2}_J$, ${\chi^2}_{J\phi}$ and χ^2_{VCD} values for the Gaussian model and the three MD force fields.

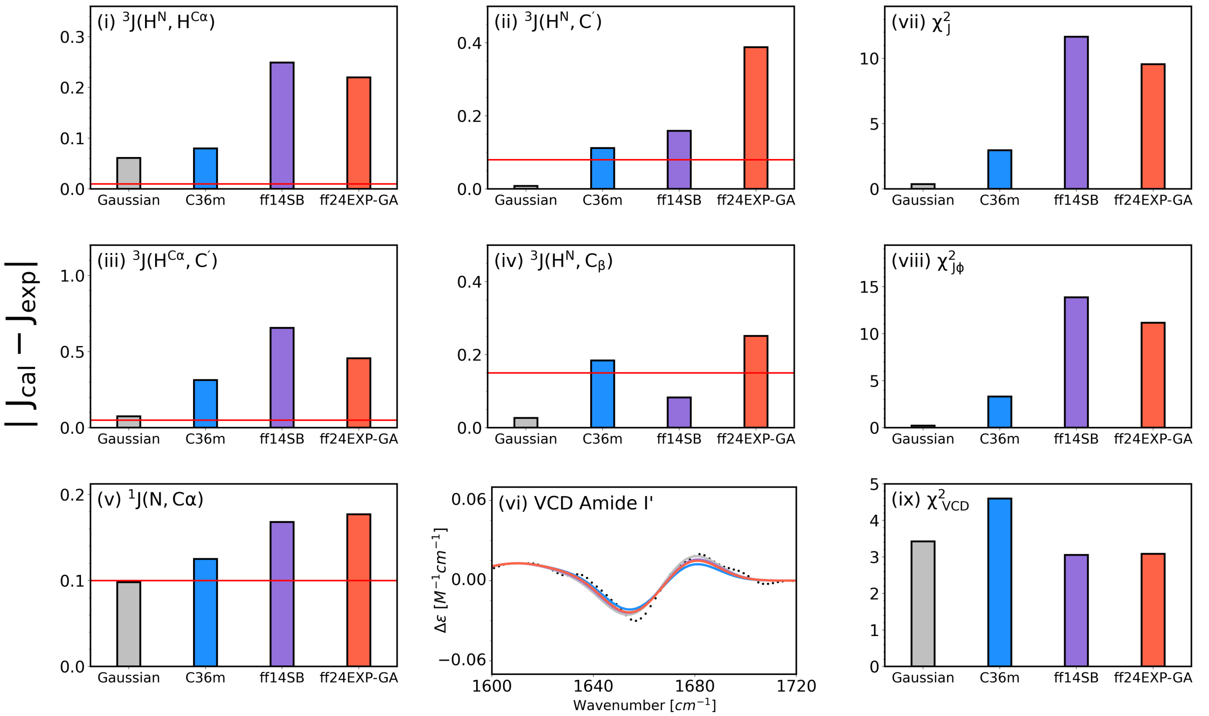

Figure S23: Comparison of experimental and computed J-coupling constants and VCD amide I' profiles of guest R in cationic GRG for the Gaussian model, CHARMM36m, Amber ff14SB and Amber ff24EXP-GA. (i-v) Absolute differences between calculated and experimental values of the five J-coupling constants for the Gaussian model and the three MD force fields. Red lines correspond to experimental uncertainties. (vi) VCD amide I' profiles computed using the Gaussian model and the three MD force fields in comparison to experimental data. (vii-ix) Reduced ${\chi^2}_J$, ${\chi^2}_{J\phi}$ and χ^2_{VCD} values for the Gaussian model and the three MD force fields.

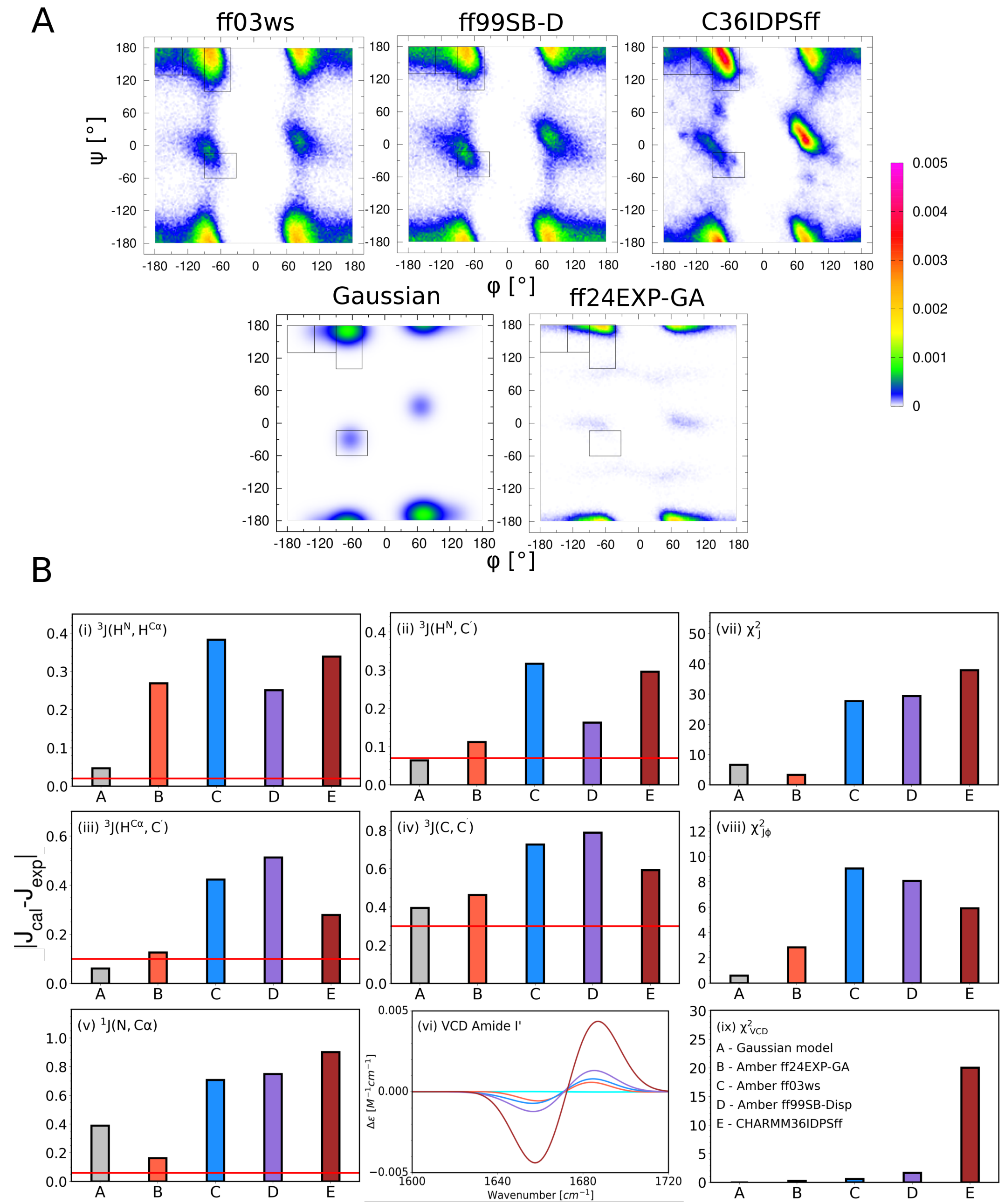

Figure S24: (A) Ramachandran distributions of guest glycine in cationic GGG for experiment-based Gaussian model (benchmark data), the parent Amber ff03ws (ff03ws), Amber 99SB-Disp (ff99SB-D), CHARMM36IDPSff (C36IDPSff), the Gaussian model (Gaussian) and newly modified Amber ff24EXP-GA (ff24EXP-GA). (B) Comparison between experimental and computed J-coupling constants and amide I' profiles of guest glycine in GGG for the Gaussian model and Amber ff14SB and Amber ff24EXP-GA. (i-v) Absolute differences between calculated and experimental values of the five J-coupling constants for the Gaussian model and the two MD force fields. Red lines correspond to experimental uncertainties. (vi) VCD amide I' profiles computed using the Gaussian model and the two MD force fields in comparison to experimental data. (vii-ix) Reduced ${\chi^2}_J$, ${\chi^2}_{J\phi}$ and χ^2_{VCD} values for the Gaussian model and the two MD force fields. ${\chi^2}_{VCD}$ values are multiplied by 10^7.

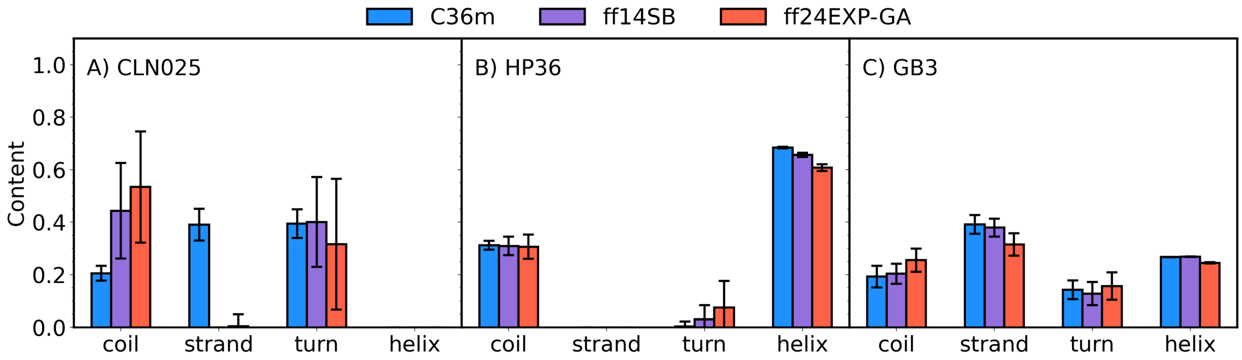

Figure S25: The average coil, turn, stand, and α-helical content of (A) CLN025, (B) HP36, and (C) GB3 derived within CHARMM36m (C36m), Amber ff14SB (ff14SB), and Amber ff24EXP-GA (ff24EXP-GA). The error bars correspond to standard deviations.

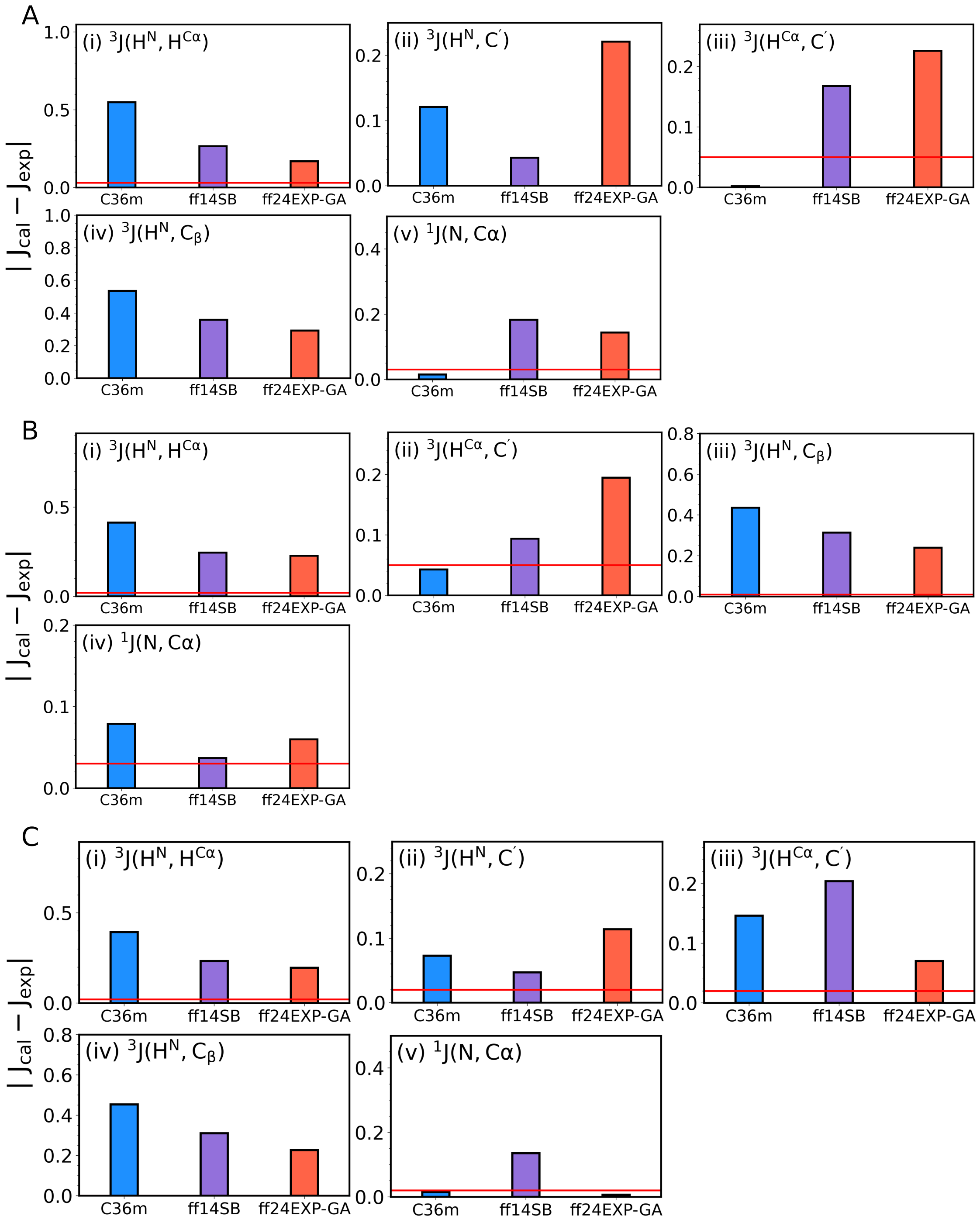

Figure S26: Absolute differences between the experimental[S4] and MD-derived J-coupling constants for residues (A) A2, (B) A3, and (C) A4 of Ala_5 obtained within CHARMM36m, Amber ff14SB, and Amber ff24EXP-GA. Red horizontal lines correspond to the reported experimental uncertainties.

References

(S1) Hu, J.-S.; Bax, A. Determination of ϕ and χ_1 angles in proteins from ^{13}C–^{13}C three-bond J couplings measured by three-dimensional heteronuclear NMR. How planar is the peptide bond? *J. Am. Chem. Soc.* **1997**, *119*, 6360–6368.

(S2) Wang, A. C.; Bax, A. Determination of the backbone dihedral angles ϕ in human ubiquitin from reparametrized empirical Karplus equations. *J. Am. Chem. Soc.* **1996**, *118*, 2483–2494.

(S3) Wirmer, J.; Schwalbe, H. Angular dependence of $^1J(N_i, C_{\alpha i})$ and $^2J(N_i, C_{\alpha(i-1)})$ coupling constants measured in J-modulated HSQCs. *J. Biomol. NMR* **2002**, *23*, 47–55.

(S4) Graf, J.; Nguyen, P. H.; Stock, G.; Schwalbe, H. Structure and dynamics of the homologous series of alanine peptides: A joint molecular dynamics/NMR study. *J. Am. Chem. Soc.* **2007**, *129*, 1179–1189

.

(S5) Heinig, M.; Frishman, D. STRIDE: a web server for secondary structure assignment from known atomic coordinates of proteins. *Nucleic Acids Res.* **2004**, *32*, W500–W502

.

(S6) Humphrey, W.; Dalke, A.; Schulten, K. VMD: visual molecular dynamics. *J. Mol. Graphics* **1996**, *14*, 33–38

.
